# Unique conformational dynamics and protein recognition of A-to-I hyper-edited dsRNA

**DOI:** 10.1093/nar/gkaf550

**Published:** 2025-06-26

**Authors:** Christoph Müller-Hermes, Valerio Piomponi, Stefan Hilber, Sam Asami, Christoph Kreutz, Giovanni Bussi, Michael Sattler

**Affiliations:** Technical University of Munich, TUM School of Natural Sciences, Bavarian NMR Center and Department of Bioscience, Garching, 85748, Germany; Helmholtz Munich, Molecular Targets and Therapeutics Center, Institute of Structural Biology, Neuherberg, 85764, Germany; Scuola Internazionale Superiore di Studi Avanzati, via Bonomea 265, Trieste, 34136, Italy; Institute of Organic Chemistry and Center for Molecular Biosciences, Innsbruck (CMBI), University of Innsbruck, Innsbruck, 6020, Austria; Technical University of Munich, TUM School of Natural Sciences, Bavarian NMR Center and Department of Bioscience, Garching, 85748, Germany; Institute of Organic Chemistry and Center for Molecular Biosciences, Innsbruck (CMBI), University of Innsbruck, Innsbruck, 6020, Austria; Scuola Internazionale Superiore di Studi Avanzati, via Bonomea 265, Trieste, 34136, Italy; Technical University of Munich, TUM School of Natural Sciences, Bavarian NMR Center and Department of Bioscience, Garching, 85748, Germany; Helmholtz Munich, Molecular Targets and Therapeutics Center, Institute of Structural Biology, Neuherberg, 85764, Germany; Cluster for Nucleic Acid Therapeutics Munich (CNATM), Munich, 80802, Germany

## Abstract

Adenosine-to-inosine (A-to-I) editing is a highly abundant modification of double-stranded RNA (dsRNA) and plays an important role in posttranscriptional gene regulation. Editing of multiple inosines by the ADAR1 enzyme leads to A-to-I hyper-editing of non-coding dsRNA, such as 3′UTRs, transposable elements, or foreign pathogenic RNAs, and is implicated in immune response and human diseases including cancer. The structural consequences of hyper-editing and its role in protein binding are poorly understood. Here, we combine solution nuclear magnetic resonance spectroscopy (NMR), biophysical methods such as small-angle X-ray scattering, and molecular dynamics simulations to study the sequence-dependent effects on conformation and dynamics of A-to-I hyper-editing for a 20-mer dsRNA and recognition of such RNAs by Endonuclease V. By comparing non-edited, single-edited, and hyper-edited dsRNA, we identify unique conformational features and extensive dynamics associated with hyper-editing, resulting in significantly increased base-pair opening. Hyper-edited dsRNA is more extended and adopts a highly dynamic ensemble of canonical and non-canonical conformations, which lead to preferential binding by Endonuclease V. Our integrated experimental and computational analysis identifies unique structural and dynamic features that are likely linked to specific protein recognition and the unique biological consequences of hyper-editing.

## Graphical Abstract

**Graphical Abstract:**
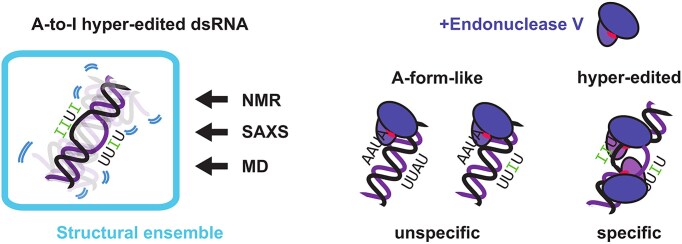

## Introduction

**Figure 1.**
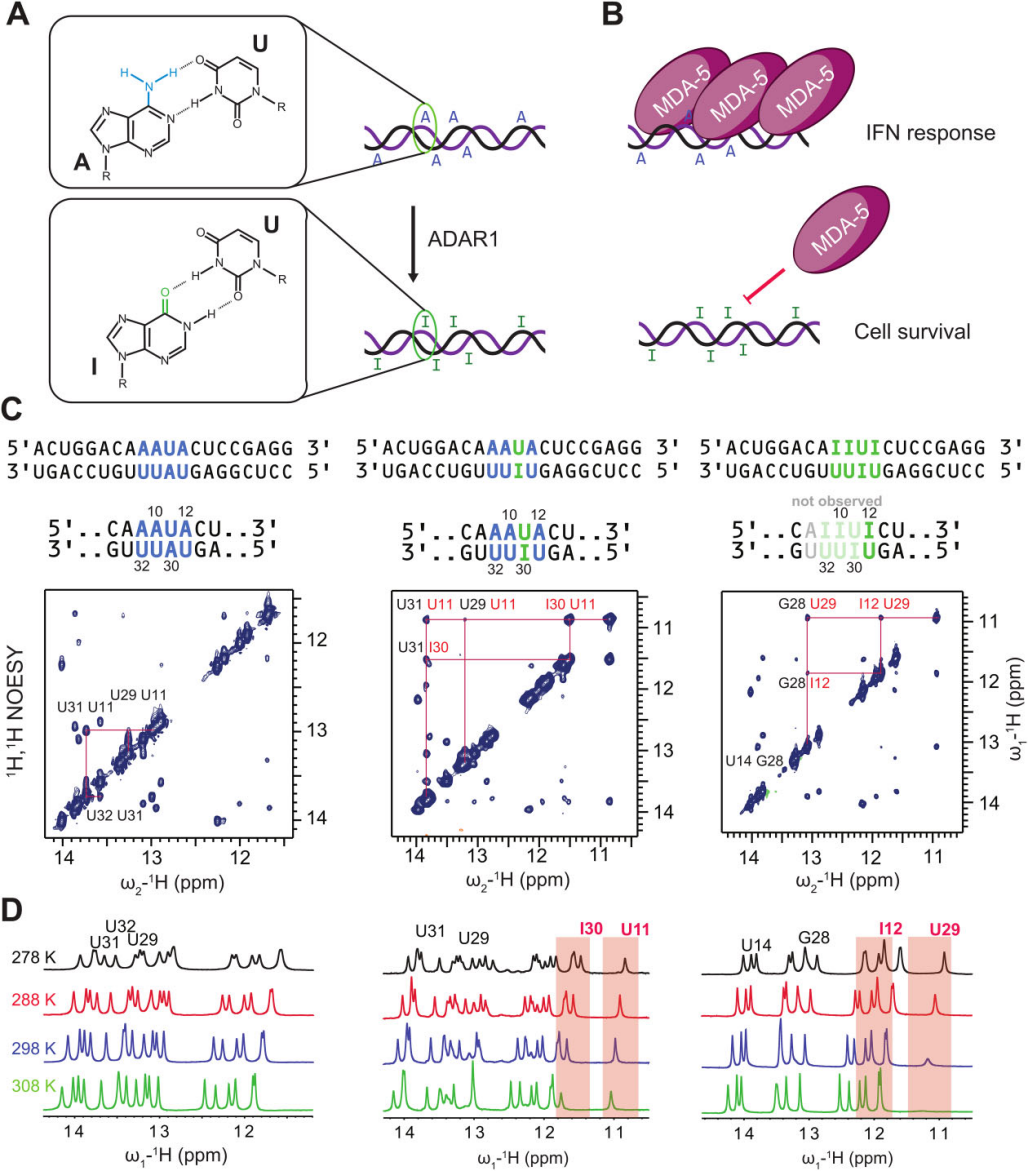
(**A**) Incorporation of inosine into dsRNA by ADAR1 leads to formation of I:U base pairs. (**B**) Cytosolic A-form RNA is recognized by MDA-5, which leads to an innate immune response, damaging the cell. Hyper-edited dsRNA cannot be recognized by MDA-5, preventing the immune response. (**C**) Model dsRNAs. ^1^H, ^1^H NOESY of the duplexes were recorded at 278 K, using a mixing time of 150 ms. For non-edited dsRNA (A-RNA) and dsRNA with a single I:U base pair (1I-RNA), a full “imino walk” was possible, indicating helicity. For hyper-edited dsRNA (I-RNA), this walk was not possible, and only the imino resonances of I12:U29 are observed. The stretch of A8-U11/I30-U33 does not show imino signals. (**D**) Upon increasing temperature I:U base pair imino resonances show line broadening, which is more pronounced in hyper-edited dsRNA.

Among various types of chemical modifications in RNA, adenosine-to-inosine (A-to-I) editing is one of the most abundant [1, 2]. A class of specific adenosine deaminases acting on RNA (ADAR) catalyzes the deamination of the exocyclic N6 amino group of adenosine, forming the non-canonical residue inosine, which features a hypoxanthine base. Depending on the member of the ADAR family, both localization and function of the editing event can vary largely. In the nucleus, site-specific A-to-I editing by ADAR2 at exonic sites leads to an A-to-G recoding, resulting in amino acid changes in the translated protein [3]. In the cytosol, ADAR1 can edit multiple sites in a double-stranded RNA (dsRNA) substrate, leading to incorporation of multiple inosines, so-called A-to-I hyper-editing [1]. Sites for hyper-editing were identified mostly in non-coding sequences like 3′UTRs of messenger RNA (mRNAs) that can form dsRNA regions, such as ALU repeats in humans or SINE/LINE in metazoans [4]. The presence of dsRNAs in the cytosol is linked to viral infection and recognized by MDA-5, which forms filaments around the A-form RNA helix and leads to an interferon response via MAVS signaling [5] (Fig. 1A). The editing activity does not appear to be sequence-specific, and levels of hyper-editing vary in different tissues, often elevated in the brain [6]. Deficiencies in ADAR1 activity are linked to autoimmune diseases like the Aicardi–Goutières syndrome (AGS), which is associated with an upregulation of interferon-stimulated genes [7]. An ADAR1 knockout was lethal in mice [8]. Moreover, the levels of A-to-I editing by ADAR1 are dysregulated in different types of cancer and cardiovascular disease [9–11]. The presence of inosine in RNA has been shown to promote the interaction with RNA-binding proteins, such as the Endonucleases Tudor-SN and Endonuclease V [12, 13]. Recognition and nucleolytic cleavage of hyper-edited RNA substrates is thought to be a signaling pathway in RNA metabolism, leading to RNA degradation [14, 15]. Endonuclease V has been reported to prefer single-stranded RNAs (ssRNAs) containing inosine residues over double-stranded substrates with single inosine sites [15, 16]. In contrast, it was suggested that hyper-edited dsRNA substrates are bound with higher affinity and cleaved with high efficiency by eukaryotic Endonuclease V homologues [13, 17]. However, the available structural studies of Endonuclease V feature complexes with palindromic substrates containing single inosine sites [18], and the molecular basis of the interaction with hyper-edited substrates remains poorly understood. Furthermore, the conformational features of such RNAs have not been studied in detail, and thus the structural effects of A-to-I hyper-editing as a driving force for protein interactions remain elusive.

The effect of A-to-I editing on dsRNA appears to be dependent on the sequence context. Incorporation of I:U base pairs into dsRNA leads to thermal destabilization, which was shown by base-pairing studies in single nucleotides as well as in melting temperature studies [19, 20]. On the other hand, A-to-I editing can also occur in A:C mismatches, which stabilizes the dsRNA by formation of I-C base pairs [21, 22]. On the single base-pair level, I:U base pairs adopt conformations similar to G:U and C:A^+^ base pairs, which show similar hydrogen bonding patterns [23]. Among these, the so-called G:U “wobble” base pair is the most stable, as the exocyclic 2-NH_2_ group of guanosine is involved in a water-mediated hydrogen bond and leads to more favorable stacking interactions [20]. The absence of this amino group could account for the instability of I:U base pairs.

A-to-I hyper-editing affects dsRNA secondary structures [24], but the exact conformational changes remain elusive. A single X-ray crystallography study of a palindromic 8-mer duplex RNA with a tandem I:U base pair has been reported and suggested an overall A-form geometry, albeit showing some local conformational effects that were similar to the ones identified for tandem G:U wobble base pairs [23]. It was observed that both wobble base-pair types G:U and I:U lead to changes in the helical twist of the dsRNA helix, resulting in an interstrand purine–purine stacking with the neighboring base pairs that leaves the pyrimidines unstacked [23, 25]. In the crystal structure, the nucleotides in I:U base pairs are base-paired, with C3′ endo ribose sugar pucker conformations. The A-form helix exhibits a slight kink, which, however, might be due to crystal packing. Solution-state NMR analysis of a hyper-edited dsRNA indicates significant deviations from an A-form geometry [26].

Here, we combine solution NMR, small-angle X-ray scattering (SAXS), and molecular dynamics (MD) simulations to study the structure and conformational dynamics of A-to-I hyper-editing for a 20-mer dsRNA with multiple A-to-I editing sites (I-RNA). This hyper-edited RNA has been linked to specific interactions with Tudor-SN and Endonuclease V proteins [12, 26, 27]. By comparing non-edited, single-edited, and hyper-edited dsRNA, we identify unique conformational features and extensive dynamics associated with hyper-editing. We show that incorporation of inosine into dsRNA leads to changes in base-pairing dynamics, where hyper-editing shows enhanced base-pair opening. We found that hyper-edited dsRNA is more extended and cannot be described as a single conformation. Our combined experimental and computational analysis demonstrates that hyper-edited dsRNA samples non-canonical conformations in a dynamic ensemble of conformations. The importance of these unique structural features and the associated conformational dynamics is reflected by a significantly increased binding affinity of Endonuclease V to hyper-edited dsRNA.

## Materials and methods

### Solid-phase synthesis of ^13^C/^15^N-labeled RNA sequences

Standard 2′-O-TBDMS RNA phosphoramidites (rA^Ac^, rC^Ac^, rG^Ac^, and rU, Chemgenes, USA) were used in combination with in-house synthesized labeled (2,8,1′,2′,3′,4′,5′-^13^C_7_)-inosine and (1,3–^15^N_2_-5-D-6,1′,2′,3′,4′,5′-^13^C_6_)-uridine RNA phosphoramidites. A controlled pore glass RNA solid support (1000 Å pore size, Chemgenes, USA) with an average loading of 40 μmol g^−1^ was used to synthesize the RNAs on an ABI 391 PCR Mate using a self-written synthesis cycle. Amidite (0.1 M) and activator (5-benzylthio-1H-tetrazole, 0.25 M) solutions were dried over freshly activated molecular sieves (3 Å) for at least 48 h before starting the solid-phase synthesis, for which following reagent mixtures were used: Cap A: acetic anhydride/lutidine/tetrahydrofuran (THF) 1/1/8, v/v/v. Cap B: THF/N-methylimidazole 86/16 v/v. Oxidation solution: 500 mg iodine dissolved in a mixture of 70 ml THF, 20 ml, pyridine, and 10 ml water. Detritylation solution: 4% dichloroacetic acid in anhydrous toluene. As soon as RNA synthesis was completed, the solid support was dried *in vacuo* for 30 min. Then standard alkaline deprotection was performed: 1 ml aqueous methylamine solution (40%) and 1 ml aqueous ammonia solution (28%–30%) were added to the solid support, and the tube was shaken vigorously and incubated at 37°C for 5 h. The solid support was filtered, then washed three times with a mixture of THF/water (1/1), and the liquid phase evaporated to dryness. The residue was dried *in vacuo* for at least 1 h before 2′-O-TBDMS deprotection was carried out. The residue from the last step was dissolved in 300 μl anhydrous DMSO, and 375 μl triethylamine trihydrofluoride was added. Then, the protection mixture was incubated at 37°C for at least 16 h. Three milliliters quenching buffer (Chemgenes, USA) were added and directly applied to a HiPrep 26/10 desalting column (GE Healthcare, Austria) using a ÄKTA start system (GE Healthcare, Austria). The crude RNA was eluted using HPLC grade water, and the RNA-containing fractions (UV detection at 254 nm) were collected in a 50 ml round-bottom flask. The solvent was evaporated, the crude RNA again dissolved in 1 ml HPLC grade water and transferred in a 1 ml Eppendorf tube. The quality of the crude RNAs was checked via anion exchange chromatography on an analytical Dionex DNAPac PA-200 column (4 × 250 mm; Eluent A: 25 mM Tris.HCl, 6 M urea, pH 8.0; Eluent B: 25 mM Tris.HCl, 500 mM sodium perchlorate, 6 M urea, pH 8.0) and at 80°C. The RNA sequences were purified in a single run by applying the crude RNA on a preparative Dionex DNAPac PA-200 column (22 × 250 mm, eluents as before). The fractions with the desired RNA were combined, and ACN was evaporated before it was loaded on a C18 SepPak cartridge (Waters, Austria) to remove HPLC buffer salts. The RNA sodium salt form was then eluted from the C18 column with water/acetonitrile (1/1, v/v), evaporated to dryness, dissolved again in 1 ml HPLC grade water, and transferred in a 1 ml Eppendorf tube for quality (analytical HPLC as before) resp. concentration determination and mass spectrometric analysis (LC-ESI mass spectrometry). Sample concentrations were determined via UV absorption at 260 nm on a NanoPhotometer (Implen). The synthesized RNAs were analyzed on Finnigan LCQ Advantage MAX ion trap instrumentation connected to a Thermo Scientific UHPLC (components: Ultimate 3000 RS Pump, Ultimate 3000 RS Autosampler, Ultimate 3000 RS Column Compartment, and Ultimate 3000 Diode Array Detector). RNA mass spectra were acquired in the negative-ion mode with a potential of −4 kV applied to the spray needle (capillary voltage: −23 V, capillary temperature: 270°C). LC: 1 μl of the dissolved RNA in 29 μl of 20 mM ethylenediaminetetraacetic acid (EDTA) solution; average injection volume: 30 μl; column: Waters xBridge C18 2.5 μm column (1.0 × 50 mm) at 30°C; flow rate: 100 μl/min; Eluent A: 8.6 mM triethylamine, 100 mM 1,1,1,3,3,3-hexafluoroisopropanol in H2O (pH 8.0); Eluent B: methanol; gradient: 0%–100% B in A within 30 min; UV detection was carried out at 260/280 nm. The correct assembly of the synthesized RNAs was confirmed by the mass data, and the RNA samples were subsequently lyophilized.

### NMR sample preparation

Unlabeled RNA oligonucleotides were purchased from Horizon Discovery Ltd. (Cambridge, United Kingdom), and selectively isotope-labeled RNA sequences were synthesized as described above. Lyophilized samples were dissolved in 25 mM sodium phosphate pH 6.4, 1 M NaCl, and 0.01 M EDTA and washed three times in a centrifugal filter unit with an MWCO of 3 kDa at 3220 × *g*, 4°C. Then, the samples were washed three times in water and flash frozen in liquid N_2_. The single strands were mixed at 1:1 molar ratio. Sample buffer (25 mM sodium phosphate pH 6.4, 25 mM NaCl) was added, and samples were lyophilized. The RNA was resuspended in either H_2_O/D_2_O 9:1 or 100% D_2_O. The strands were hybridized by incubation at 95°C for 5 min, followed by an incubation at 75°C for 5 min in a thermo-block (Eppendorf SE, Hamburg, Germany). The thermo-block was switched off, and the sample was slowly cooled down to room temperature. For NMR measurements, samples were transferred into a 3 mm NMR tube (Norell Inc., Landisville, USA) at 250–500 μM concentration.

### NMR spectroscopy

All experiments were recorded on 600, 800, or 900 MHz Bruker NMR spectrometers with AVIII consoles, equipped with TCI cryogenic probes. Spectra were processed in TopSpin v3.5pl6 and analyzed in CCPNMR v2.4.2.

#### Chemical shift assignments

For the assignment of imino resonances, spectra were recorded at 278 K. Homonuclear ^1^H,^1^H-NOESY experiments were recorded for A-, 1I-, and I-RNA samples using a mixing time of 150 ms. Water suppression was achieved by WATERGATE with water flip-back [28–30]. Nitrogen resonances were assigned by measuring SOFAST-HMQC experiments [31] at natural abundance of ^15^N. SOFAST-HMQC experiments were used to identify the type of nucleotide based on the ^15^N chemical shift. The ^1^H,^1^H-NOESY was used to generate sequential links based on inter-nucleotide imino-imino proton distances (“imino walk”). For the non-exchangeable resonances of the RNA backbone, samples in 100% D_2_O were used. ^1^H,^1^H-NOESY using excitation sculpting for water suppression [32] were recorded for A- and I-RNA samples using mixing times of 150 and 300 ms. The distances of the H1’ to the H6/H8 of its own nucleotide as well as the preceding one were used to generate sequential assignments, a so-called “anomeric–aromatic walk.” To resolve overlap in the ^1^H,^1^H-NOESY, a 3D NOESY-^1^H,^13^C-HMQC experiment [30, 33] with pre-saturation pulse for water suppression was recorded on a selectively ^13^C-labeled I-RNA sample. The NOESY mixing time was 150 ms. For the labeled residues in I-RNA, ribose resonance assignments were achieved based on 3D H(C)CH-TOCSY and H(C)CH-COSY experiments [30, 33].

#### Hydrogen exchange experiments

A series of 1D proton WATERGATE water flip-back experiments was recorded at 278, 288, 298, and 308 K on unlabeled A-RNA, 1I-RNA, and I-RNA samples at a concentration of 500 μM. 1D CLEANEX-PM experiments [34] were recorded at 287 K on a 600 MHz NMR spectrometer, using a 3-9-19 water gate sequence with a selective flip-back pulse for water suppression and ^15^N composite pulse decoupling (based on pulse sequence zgcxgp19). Build-up experiments were acquired using CLEANEX mixing times of 5, 10, 30, 40, 60, 80, and 100 ms. Spectra were analyzed in TopSpin v3.5pl6. Peak intensities were normalized to a reference measurement, which did not include the CLEANEX element. The resulting build-up curves were fitted to the following model in Origin Pro version 9.0G:


\begin{equation*}
\frac{{I\left( {{{\tau }_{{\rm mix}}}} \right)}}{{{{I}_{{\rm ref}}}}} = \ \frac{{{{k}_{{\rm ex}}}}}{{{{R}_{1,A}} + {{k}_{{\rm ex}}} + {{R}_{1,W}}}}\left[ {{\mathrm{exp}}\left( { - {{R}_{1,W}}{{\tau }_{{\rm mix}}}} \right) - {\mathrm{exp}}\left( { - \left( {{{R}_{1,A}} + {{k}_{{\rm ex}}}} \right){{\tau }_{{\rm mix}}}} \right)} \right],
\end{equation*}

*k*_ex_ is the apparent hydrogen exchange rate, *R*_1*,A*_is the longitudinal relaxation rate of the respective imino proton, and *R_1,W_*is the longitudinal relaxation rate of the water proton. The parameters *k*_ex_ and *R*_1,*A*_ were set freely; *R*_1,*W*_ was determined using a saturation recovery experiment on a buffer sample without RNA, as described in [35].

#### Ribose conformation

^13^C chemical shifts were used to calculate the canonical coordinates *can1* and *can2* as described [36]:


\begin{eqnarray*}
can1 = 0.179{{\delta }_{C1^{\prime}}} - 0.225{{\delta }_{C4^{\prime}}} - 0.0585{{\delta }_{C5^{\prime}}}
\end{eqnarray*}

\begin{eqnarray*}
can2 = - 0.0605\left( {{{\delta }_{C2^{\prime}}} + {{\delta }_{C3^{\prime}}}} \right) - 0.0556{{\delta }_{C4^{\prime}}} - 0.0524{{\delta }_{C5^{\prime}}}
\end{eqnarray*}

Scalar couplings ^3^*J*(H1′,H2′) were extracted from a 3D forward-directed HCC-TOCSY-CCH-E.COSY experiment [37] on a selectively ^13^C-labeled I-RNA sample. The values were used to assess the ribose sugar pucker conformations based on the Karplus equation in [38], in which a C3′ endo ribose has a ^3^*J*(H1′,H2′) value of 0–1 Hz and a C2′ endo ribose has a value of 10–12 Hz.

#### NMR relaxation experiments

On-resonance ^15^N *R*_1ρ_ relaxation dispersion (RD) experiments [39] were recorded on an 800 MHz spectrometer at 282 and 287 K on a 500 μM I-RNA sample with selectively ^15^N1-labeled inosine residues. Spin lock fields of 100, 125, 150, 175, 200, 250, 300, 350, 400, 450, 500, 550, 600, 700, 800, 900, 1000, 1100, 1200, 1300, 1400, 1500, 1600, 1800, and 2000 Hz were used. For each spin lock field, 11 relaxation delays of 0, 2.5, 5, 5, 7.5, 10, 15, 20, 30, 40, and 50 ms were recorded. Each decay curve was fitted using a single exponential decay using a Python script (provided by Dr Lorenzo Baronti and Dr Katja Petzold). ^13^C CPMG RD experiments [40] were recorded on a 500 μM sample of I-RNA with selective ^13^C labeling on a 800 MHz NMR spectrometer at 308 K. A pseudo-3D experiment was recorded using 13 CPMG fields: 50, 100, 150, 200, 250, 300, 400, 500, 600, 700, 700, 800, and 1000 Hz. In addition, a reference spectrum without the CPMG pulse train was recorded. The value of *R*_2,eff_ was calculated by


\begin{eqnarray*}
{{R}_{2,{\rm eff}}} = \ - \frac{1}{{{{T}_{{\rm CPMG}}}}}{\mathrm{ln}}\left( {\frac{{{{I}_{{\rm CPMG}}}}}{{{{I}_0}}}} \right).
\end{eqnarray*}

On resonance ^13^C *R*_1ρ_ RD experiments [41] were recorded for ribose C1′ atoms in a 500 μM sample of I-RNA with selective ^13^C labeling at 800 MHz and 308 K. ^13^C spin lock fields of 100, 150, 200, 250, 300, 350, 400, 450, 500, 550, 600, 700, 800, 900, 1000, 1500, 2000, and 2500 Hz were used. At each spin lock field, a decay curve using relaxation delays of 0, 10, 20, 30, 40, and 50 ms was measured. To control sample heating, a heat compensation element was used in the pulse program as described in [41].

{^1^H}-^13^C heteronuclear nuclear Overhauser effect experiments (hetNOE) [42] were recorded at 298 K and 800 MHz, using a recycling delay of 5 s to reduce saturation. ^13^C *R*_2_ experiments [43] were recorded at 298 and 308 K and 800 MHz, using relaxation delays of 22.4, 44.8, 67.2, 89.6, 112.0, 134.4, 156.8, and 179.2 ms. For both experiments, spectra were analyzed using CCPNMR analysis 2.4.2. ^13^C *R*_2_ values were extracted from fitting single exponential decay functions. {1H}-^13^C hetNOE values were calculated from the signal intensity ratio in the saturated and non-saturated experiments.

#### NMR structure calculation

NOE distance restraints were extracted from 2D ^1^H,^1^H-NOESY and 3D NOESY-HMQC spectra. For 2D spectra, NOE restraints were referenced by a pyrimidine H5–H6 cross peak. For the 3D NOESY, NOEs were calibrated by a ribose H1′–H2′ signal for a residue adopting C3′ endo conformation (U23). The NOE restraints then were binned into categories strong (1.8–3.3 Å), medium (2.0–4.5 Å), and weak (3.3–5.1 Å). For the central IIUI/UIUU motif of I-RNA, NOE restraints were taken from 3D experiments to avoid signal overlap. In order to account for ^13^C relaxation effects during heteronuclear magnetization transfers, the restraints were compared to the corresponding NOE restraints derived from the 2D ^1^H,^1^H-NOESY experiment. For residues of the flanking regions, non-overlapping cross peaks from 2D spectra were analyzed and compared to a spectrum recorded under the same conditions on a non-modified A-RNA sample. As the restraints were matching, they were used as semi-empirical restraints and were generalized for the entire flanking part, restraining it in A-form conformation. Ribose moieties were restrained in C2′ endo if ^3^*J*(H1′,H2′) > 3 Hz and *can1* < −6.25 ppm. Otherwise, the ribose moieties were restrained in C3′ endo conformation. The backbone dihedral angles *α*, *β*, *γ*, *δ*, and *ϵ* were loosely restrained in an A-form conformation for the central motif (using an error of ±20°), whereas the flanking stem regions were restrained tighter in A-form conformation (using an error of ±10°). As all intra-nucleotide H8/H6-H1′ cross peaks were weak and of similar intensities for all nucleotides, the *χ* angle was restrained in *anti* for all nucleotides. For base pairs with observable imino resonances, hydrogen bond restraints were introduced and planarity restraints with a high weight. For base pairs with non-observable imino resonances, a low weight was used for the planarity restraints. Extended structures were generated and folded using a simulated annealing protocol in XPLOR-NIH [44, 45] using an RNA-ff1 force field [46]. A protocol was used which consists of an initial high temperature step with small force constants, followed by a simulated annealing step in which the temperature was lowered and the force constants increased. The final step was an energy minimization in torsional angle space. 100 structures were generated. The lowest energy structure was subjected to refinement using a similar protocol. 100 structures were generated. For none of these structures, NOE violations (<0.5 Å) and the majority shows no violation of torsional angle restraints (<5 Å). The statistics are shown in Table 1.

**Table 1.** Structural statistics of A-RNA and I-RNA

	I-RNA*	A-RNA
Total number of restraints	521	480
NOEs	191	140
- *Intra-residue*	56	0
- *Inter-residue*	135	140
Torsional angles^a^	248	250
Planarity^a^	40	40
H-bonds^a^	42	50
NMR ensemble^a^		
RMSD NOE restraints (Å)	0.067 ± 0.001	0.055 ± 0.001
NOE violations (>0.5 Å)	0	0
Torsion violations (>5°)	0.3 ± 0.7	0
RMSD of the mean coordinates (Å)	0.309 ± 0.01	0.241 ± 0.009
RMSD from ideal geometry		
- *bond lengths (Å)*	0.002 ± 0.000	0.001 ± 0.000
- *bond angles (°)*	0.660 ± 0.006	0.635 ± 0.006
Total energy (kcal/mol)	−1050.50 ± 15.59	−1436.74 ± 10.17

*Hypothetical I-RNA structure assuming complete C2′ endo sugar puckers for residues 10, 11, 30, 31, and 32.

^a^Torsion angle restraints for *α*, *β*, *γ*, *δ*, *ϵ*, ζ, and *χ* were applied for residues 1–40, see “Materials and methods” section.

^b^Planarity restraints were described for residue 1–40 in both RNAs.

^c^H-bond restraints were applied for residues 1–40 in A-RNA, and residues 1–7, 12–20, 21–29, and 34–40 in I-RNA.

^d^10 lowest energy structures from 100 calculated.

#### Circular dichroism spectroscopy

Circular dichroism (CD) measurements were performed on a Jasco J-1500 CD spectropolarimeter (Jasco Inc., Easton, USA) using a quartz cuvette with a 10 mm path length. Twenty micromolar samples of unmodified A-RNA and hyper-edited I-RNA in 25 mM sodium phosphate pH 6.4, and 25 mM NaCl were prepared. To ensure duplex hybridization, a denaturing-renaturing program was run prior to the CD measurement, consisting of a heat-up step until 95°C and subsequent cool-down step to 20°C with a heating/cooling rate of 1°C/min. Each spectrum was recorded at 20°C using wavelengths from 190 to 320 nm with a data pitch of 0.1 nm, a bandwidth of 2 nm, a dwell time of 1 s, and by accumulating five measurements.

#### Small-angle X-ray scattering

SEC SAXS measurements of A-RNA and I-RNA were performed at the BM29 beamline at the ESRF (Grenoble, France) using a Pilatus 3D detector. The samples were subjected to a 5 ml Superdex S300 5/150 GL chromatography column (Cytiva, Marlborough, USA) with a flow rate of 0.2 ml/min. Frames were measured with an exposure time of 2 s at 20°C with the wavelength *λ* = 0.09919 nm. A total of 500 frames was recorded. Frames of eluted peaks with a stable radius of gyration were selected, and buffer subtraction was performed using CHROMIXS [47]. The modulus of the scattering vector *q* = (4π/λ)sinθ (scattering angle 2θ) was used to express the scattering intensities. The data were analyzed using PRIMUS [47]. For validation, the molecular weight of the RNAs was calculated from a Guinier plot, which was also used to extract the radius of gyration (*R*_g_) of the samples. The SAXS data were used to validate structural models from NMR structure calculations using the CRYSOL function in the ATSAS v.3.2.2 package [47]. Frames from MD trajectories were subjected to CRYSOL fitting. Back-calculated SAXS profiles were averaged and used either all or as subsets, which were obtained from a clustering, to fit the experimental SAXS data for validation.

#### MD simulations

Starting structures for MD simulations were built using the proto–Nucleic Acid Builder [48]. Simulations were performed with GROMACS 2020 [49], using TIP3P water [50], the AMBER force field for nucleic acids [51–53], plus partial charges taken from modrna08 for hypoxanthines [54]. The sugar and phosphate moieties of inosines were parameterized using the standard AMBER force field. We note that the bonded and non-bonded types for hypoxanthines were also taken from the AMBER force field, resulting in identical parametrization for bonded interactions. In principle, the torsional potential on the glycosidic bond angle could have been reparametrized using the procedure used for the χOL3 parametrization [52]. For simplicity, we decided to directly use the parameters of guanosine, which is the nucleoside with the electrostatic potential expected to be the most similar to hypoxanthine. The systems were first energy minimized and subjected to a multi-step equilibration procedure. In the production runs, a thermostat [55] and a barostat [56] were used to maintain the temperature at 300 K and pressure at 1 bar. The inosines (adenosines) double-strand system had 53 868 (53 944) atoms, including sodium and chloride ions [57], resulting in a neutralized system with a salt concentration of 0.1 M. Simulations were integrated in a replica exchange with collective-variable tempering (RECT) scheme [58] in order to ensure exhaustive sampling with respect to sugar puckering conformations. RECT was applied to the sugar pseudorotation Zx [59] variables of the 24 central nucleotides. In our scheme, 8 replicas were used, in which well-tempered metadynamics [60] were performed with a bias factor that is scaled along the replica ladder with a geometric series ranging from 1 to 5. Metadynamics was implemented in the simulations using the PLUMED package [61], and exchanges within replicas were proposed every 100 steps.

After production, trajectories were reweighted to match experimental observables following the maximum entropy (ME) principle [62]. ME was applied on inosine dsRNA for 9 ^3^*J*(H1′,H2′) scalar couplings and the averaged radius of gyration squared. Davies parameters [38] were used to back-calculate ^3^*J*(H1′,H2′) scalar couplings from trajectories. We collected three sets of simulations: (i) adenosine dsRNA (A-RNA) (350 ns per replica); (ii) inosine ds-RNA (I-RNA) (366 ns per replica); and (iii) I-RNA (350 ns per replica); for a total of (350 + 366 + 350 ns) × 8 = 8.528 μs. The same enhanced sampling scheme was used in the three simulations. In addition, in case (iii), additive restraints were used, acting on the 9 ^3^*J*(H1′,H2′) scalar couplings, in order to increase the sampling of conformers compatible with the NMR data. The pre-factors for these restraints were obtained by analyzing with ME the set of simulation in (ii). The restraints were scaled along with the replica index by dividing for the respective bias factor. Trajectories were clustered using the quality threshold algorithm [63].

#### Protein expression and purification

Wild-type *Escherichia coli* EndoV (residues 1-223) was expressed as a construct with an N-terminal His-tag and a TEV cleavage site. LB medium with 30 μg/ml kanamycin was inoculated with *E. coli* BL21(de3) containing pET-M11-EcEndoV (residues 1-223). The culture was grown at 37°C, 280 rpm in an incubation shaker until an OD_600_ of 0.6–0.8 was reached. Protein overexpression was induced using 0.25 mM isopropyl β-thiogalactopyranoside (IPTG). Growth was continued at 20°C, 180 rpm for 20 h. Cells were harvested by centrifugation at 7000 × *g*, 4°C for 20 min. The pellet was dissolved in lysis buffer (50 mM Tris pH 8.0, 300 mM NaCl, 10 mM imidazole, 5 mM β-mercaptoethanol) and protease inhibitor mix, and DNase and lysozyme were added. The suspension was incubated on ice for 30 min. Cells were lyzed by passing them two times through a French pressure cell at 15 k psi. The lysate was clarified by centrifugation at 4°C for 1 h. The clarified lysate was passed twice through a gravity flow nickel IMAC column. The column was washed two times with 10 column volumes. Then, the bound protein was eluted using 20 ml of EndoV elution buffer (50 mM Tris pH 8.0, 300 mM NaCl, 250 mM imidazole, 5 mM β-mercaptoethanol). The His-tag was cleaved off by adding TEV protease (ratio 1:150) and the solution was dialyzed against 2 L EndoV lysis buffer using SnakeSkin (Thermo Fisher Scientific, Rockford, USA) dialysis bags (MWCO 10 kDa) at 4°C overnight. The protein solution was passed through a gravity flow nickel IMAC column twice, in which the column flowthrough was collected. The flowthrough was concentrated using an Amicon (Merck KGaA, Darmstadt, Germany) centrifugal filter unit (MWCO 10 kDa) until a volume of 1–2 ml was reached. The solution was loaded on a Superdex S85 column (Cytiva, Marlborough, USA), attached to an ÄKTA chromatography system (Cytiva, Marlborough, USA), and equilibrated using a storage buffer (25 mM sodium phosphate, pH 6.5, 300 mM NaCl, 5 mM dithiothreitol (DTT)). The fractions containing pure protein were concentrated, and aliquots were flash frozen in liquid N_2_.

#### Enzymatic cleavage assay

RNA samples were prepared as 1 μM stocks in 40 mM Tris pH 7.5, 50 mM sodium chloride, 1 mM DTT, and 5 mM manganese chloride or magnesium chloride. Alternatively, 25 mM sodium phosphate pH 6.5, 50 mM NaCl, 1 mM DTT, and 5 mM manganese chloride or magnesium chloride were used. Per reaction, 1 pmol of RNA was used in a reaction volume of 10 μl. EndoV was added at increasing concentrations of 0, 70, 140, 210, and 280 nM from a 7 μM stock solution in EndoV cleavage buffer. Reactions were incubated at 37°C for 30 min in a heat block. Then, reactions were stopped by addition of denaturing loading dye, and a 20% denaturing acrylamide gel was run in Tris/borate/EDTA (TBE) buffer at 10 W for 25 min. The gel was stained using 0.004% SYBR Gold solution in TBE buffer for 5 min and then visualized using UV detection in a gel documentation device.

#### Electrophoretic mobility shift assay

RNA samples were prepared as 2 μM stocks in the respective electrophoretic mobility shift assay (EMSA) buffer condition (25 mM sodium phosphate pH 6.5, 50–300 mM sodium chloride, 1 mM DTT, 5 mM magnesium chloride). EndoV samples in EndoV storage buffer were diluted with EMSA buffer to yield stock solutions of 1 and 7 μM. EMSA reactions were prepared in 10 μl, using 2 pmol of RNA per reaction and protein/RNA ratios of 0, 0.25, 0.5, 1, 2, 3.5, 7, 10.5, and 21. The samples were incubated on ice for 30 min. Then glycerol was added from a 30% glycerol stock to reach a final concentration of 10%. The samples were loaded onto a 12% native acrylamide gel and the gel run in TBE buffer for ca. 20 min at 10 W and at room temperature. The gel was stained using a 0.004% SYBR Gold solution in TBE buffer for 5 min and then visualized using UV detection in a gel documentation device (Cytiva, Marlborough, USA).

## Results

### Assignment of IU base-pair resonances

Unlabeled 20-mer dsRNA samples were used to analyze the base-pairing in non-edited (A-RNA), single- (1I-RNA), and hyper-edited (I-RNA) dsRNA harboring one and four I:U base pairs, respectively. In the inosine-containing sequences, imino proton NMR signals around 11 and 12 ppm were observed, indicative of non-canonical base pairs, such as I:U base pairs (Fig. 1C). The imino signal at 12 ppm was assigned to inosine, based on the downfield ^15^N chemical shift value of ca. 170 ppm, indicative for inosine [64, 65] (Supplementary Fig. S1). Imino resonances could be assigned sequentially using ^1^H, ^1^H-NOESY spectra (Fig. 1C). Apart from the terminal base pairs, a complete “imino walk” is possible in the non-edited A-RNA and 1I-RNA, suggesting an overall A-form geometry. In the hyper-edited I-RNA, the imino walk is possible in the regions flanking the central hyper-edited motif. Imino NMR signals for A8:U33 to U11:I30 are not observed. The imino signals corresponding to I:U base pairs were thus assigned to the base-pair I12:U29. For the stretch from base-pair A8:U33 until U11:I30, no imino signals are observed for I-RNA, as we reported previously [26]. These data indicate that the boundaries of the central motif have a higher tendency to form an A-form helix, while the central hyper-edited region exhibits significant conformational dynamics. Addition of Mg^2+^ leads to changes in the imino resonances of the entire sequences, but the I:U base pairs do not act as preferred Mg^2+^ binding sites and no stabilization of the hyper-edited motif is observed (Supplementary Fig. S2).

NMR signals of non-exchangeable protons were assigned for A- and I-RNA using ^1^H, ^1^H-NOESY, and -TOCSY spectra. Due to overlap in the anomeric–aromatic region, 3D NOESY-^1^H,^13^C-HMQC, H(C)CH-TOCSY, and H(C)CH-COSY experiments were recorded on selectively ^13^C-labeled samples of I-RNA to resolve signals (Supplementary Fig. S3). Using the 3D experiments, the anomeric–aromatic NOE walk was possible in the central motif of I-RNA. However, the NOEs connecting the residues show variable intensities, with particularly weak NOE correlations observed for I10H8-I9H1′ and I30H8-U29H1′ (Supplementary Fig. S5A). This suggests that the geometry of I-RNA deviates from A-form geometry but retains an overall helical structure.

The more canonical behavior of the motif toward I12:U29 may be rationalized by two considerations: (i) The flanking C13:G28 base pair may act stabilizing on the I12:U29 base pair, while the A8:U33 base pair flanking I10:U32 on the other end of the hyper-edited region may not have this effect. In order to test this hypothesis, we replaced A8:U33 by a corresponding G:C base pair, but still could not detect additional I:U imino proton resonances (Supplementary Fig. S4). (ii) Altered stacking interactions between the I:U/I:U and U:I/I:U motifs present in the central part might differentially influence the I:U base pair stability. To test this hypothesis, base-pair I9:U32 was flipped to yield I32:U9. By this, these I:U base pairs have the same arrangement as seen for U11:I30 and I12:U29 with inosines in opposite strands. Indeed, in this context additional imino resonances in the regions corresponding to I:U base pairs are observed (Supplementary Fig. S4). The I:U base pair is thought to adopt wobble pair geometry, which affects the helical twist by stacking with neighboring bases. Thus, incorporation of multiple I:U base pairs affects the backbone conformation of dsRNA. As the arrangement of the base pairs can have significant effects on stability, nearest neighbor effects present in the U:I/I:U motif might to some extent compensate for the destabilizing effects of inosine editing.

### A-to-I hyper-editing pairs leads to increased base-pair opening rates

**Figure 2.**
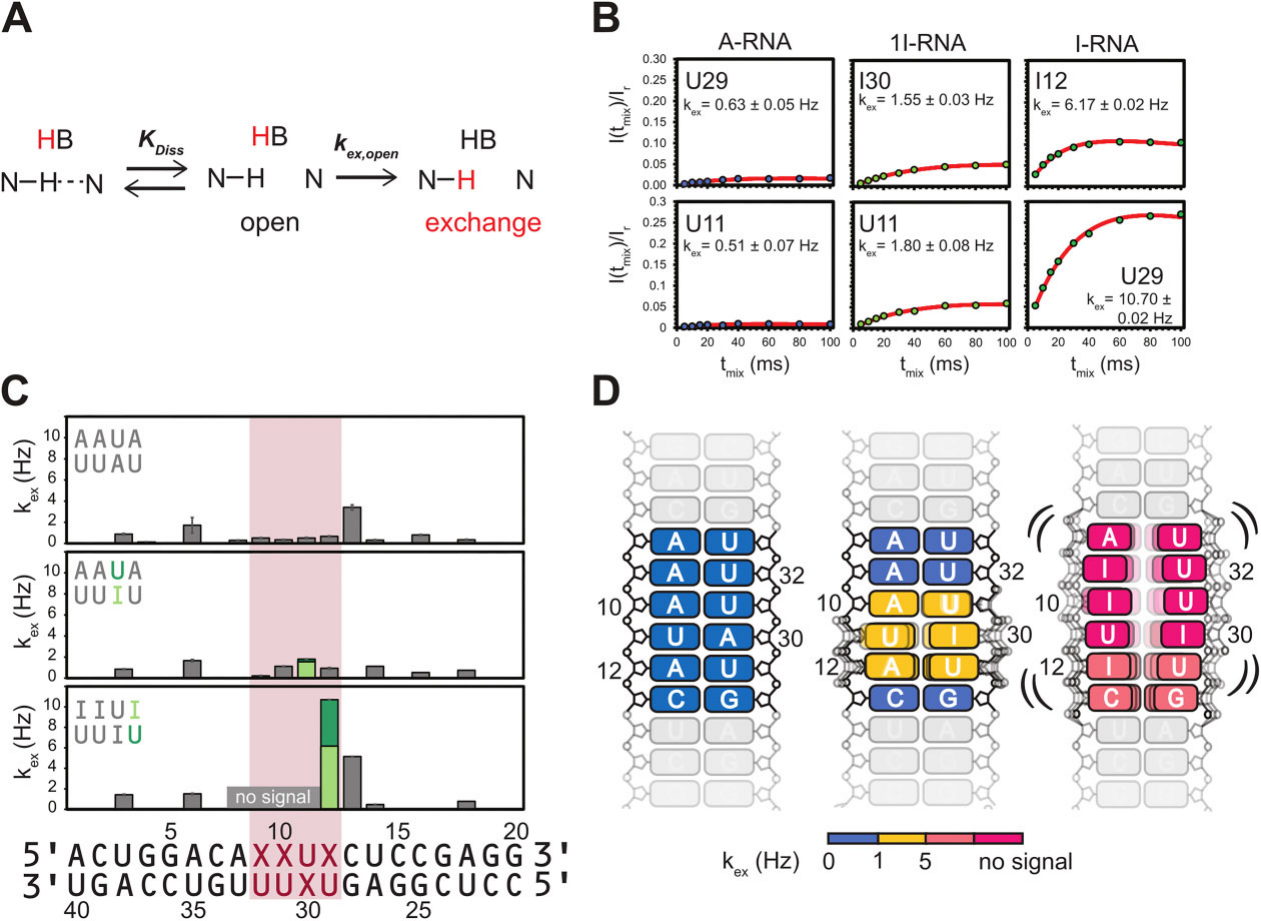
(**A**) Mechanism of solvent exchange between a base catalyst (i.e. H_2_O or a buffer component) and an imino proton in a base pair. The base pair shows an equilibrium between open and closed states. Exchange happens from the open state. (**B**) CLEANEX-PM build-up curves of imino signals in the central motif of A-, 1I-, and I-RNA. Exchange parameters *k*_ex_ are indicated. (**C**) Column plot of the *k*_ex_ values of all signals in the three dsRNAs. The central region is indicated by a red box. (**D**) Cartoon representation of base-pair opening in the three dsRNAs. The values of *k*_ex_ are color-coded. Signals not observed in the 1D spectra are shown in dark red, indicating predominantly open states.

When measuring 1D imino spectra at increasing temperature, we observed selective line broadening of the resonances of I12:U29 in I-RNA (Fig. 1D), indicating exchange broadening due to conformational exchange. In contrast, ^15^N *R*_1ρ_ RD experiments show flat RD profiles (Supplementary Fig. S6), suggesting the absence of μs-ms time scale dynamics [66–68]. Next, to characterize individual base pair stabilities, we measured hydrogen exchange of base-pair imino protons (Fig. 2A) with bulk water protons using 1D CLEANEX-PM experiments (Supplementary Fig. S5). For the non-edited A-RNA duplex exchange rates, *k*_ex_ < 1 Hz are obtained for the central four base pairs, which are of the same magnitude as the *k*_ex_ values of the flanking parts (Fig. 2B and C). For 1I-RNA and I-RNA, larger values of *k*_ex_ are observed compared to A-RNA, which are 3-fold and up to 20-fold increased for 1I-RNA and I-RNA, respectively (Fig. 2B and C). For the central motif, incorporation of I:U base pairs increases base-pair opening also for adjacent base pairs, while not affecting the flanking stem regions in the dsRNAs (Fig. 2C). For I-RNA imino proton signals of the central I:U base pairs, except for I12:U29, are not observable in 1D proton spectra at pH 6.4 and 278 K (Fig. 1C), while at a lower pH of 5.8, additional signals are detectable in the region of I:U base pairs, indicating reduced exchange and base pair opening (Supplementary Fig. S6D). However, these signals could not be assigned by NOESY experiments due to fast solvent exchange. These data indicate that the presence of I:U base pairs induces slow base-pair dynamics at millisecond to second time scales (Fig. 2D).

### A-to-I hyper-editing promotes C2′ endo sugar pucker conformation

**Figure 3.**
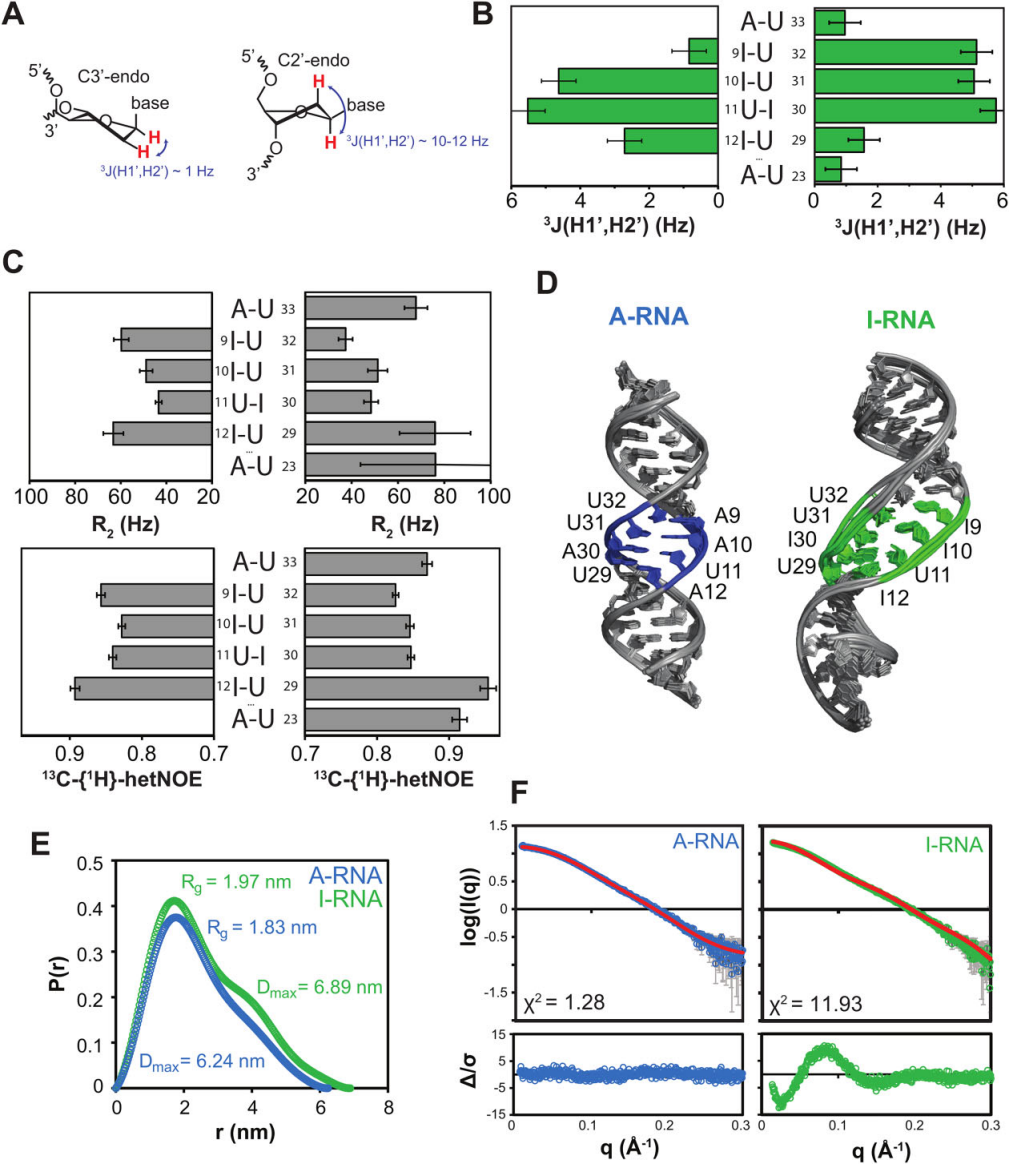
(**A**) C3′ endo and C2′ endo conformations lead to different values of ^3^*J*(H1′,H2′). (**B**) ^3^*J*(H1′,H2′) values for the residues of the central motif in I-RNA. (**C**) ^13^C R_2_ rates and {^1^H}-^13^C hetNOE values for the aromatic C8/C6 in the central motif of I-RNA. (**D**) NMR-derived structural models of A-RNA (blue) and I-RNA (green). (**E**) Pairwise distance distributions of A-RNA (blue) and I-RNA (green); *R*_g_ and *D*_max_ values are indicated. (**F**) SAXS profiles of A-RNA and I-RNA, including fits and residuals for structural models.

We measured ^3^*J*(H1′,H2′) scalar couplings to investigate the ribose sugar pucker conformation in the hyper-edited I-RNA. The preferred sugar pucker conformations in dsRNA and single-stranded RNAs are C3′ endo and C2′ endo conformations, respectively. Canonical A-form dsRNA features C3′ endo ribose conformations, for which ^3^*J*(H1′,H2′) < 1 Hz. The C2′ endo conformation, which is commonly found in single-stranded RNA or B-form helices, exhibits large values of ^3^*J*(H1′,H2′) of 10–12 Hz (Fig. 3A). Residues at the edges of the central motif (I9, U29) have ^3^*J*(H1′,H2′) values of ∼1–2 Hz, comparable to U23, which is located in one of the flanking helical regions. Thus, these residues adopt the canonical C3′ endo conformation. For residues I10, U11, I12, I30, U31, and U32 larger ^3^*J*(H1′,H2′) couplings were measured up to ≈ 6 Hz. These values are smaller than expected for a pure C2′ endo conformation, indicating that the sugar puckers in the central motif adopt dynamic populations of C2′ endo and C3′ endo conformations. Surprisingly, very different *J*-couplings are observed for nucleotides involved in the same base pair, such as I9 and U32 (0.84 and 5.14 Hz, respectively). Further analysis of the ribose conformation is based on ^13^C chemical shifts for which canonical coordinates, can1 and can2, are correlated with the ribose sugar pucker [36, 69] (Supplementary Fig. S7). These data are consistent with the ^3^*J*(H1′,H2′) values and indicate population of C2′ endo conformations for the central base pairs. The can1/can2 values also reflect the different behavior of I9 and U32.

Next, we probed dynamics on ps–ns time scales with ^13^C transverse relaxation rates (*R*_2_) and {^1^H}-^13^C heteronuclear NOE values for aromatic C8/C6 atoms (Fig. 3C). These data show that nucleotides flanking the central motif exhibit higher *R*_2_ and hetNOE values, indicating more rigid conformations. Interestingly, residues with larger ^3^*J*-couplings, suggesting increased populations of C2′ endo sugar puckers (I10, U11, I30, U31, and U32), show greater flexibility on the ps–ns time scales. Notably, some of the residues adopt C3′ endo conformations and are less flexible, e.g. U33 and I9, but still do not show detectable imino resonances. Other residues, such as I12 and U29, show increased populations of C2′ endo sugar puckers and have detectable imino resonances. This suggests that the base-pair opening dynamics (indicated by non-observable imino protons) are not correlated with fluctuations in the sugar puckering. To investigate the potential exchange between the C3′ endo and C2′ endo sugar puckers at μs–ms time scales, we recorded on-resonance ^13^C *R*_1ρ_ RD experiments [41] for the ^13^C1′ atoms of residues I9, U11, I12, and I30 (Supplementary Fig. S8C). Flat profiles indicate the absence of significant conformational dynamics on this time scale. Similarly, flat profiles are observed for aromatic C8/C6 atoms in ^13^C CPMG RD experiments, which we recorded to examine potential base flipping out of the helix on the μs–ms time scale (Supplementary Fig. S8B). We note that the absence of μs–ms time scale dynamics does not exclude the presence of exchange on a slower time scale, e.g. at milliseconds to seconds. Based on NMR data, we could not assess whether the C3′–C2′ endo conformational changes in the hyper-edited region are uncorrelated or concerted, i.e. with ribose moieties in the central motif changing the sugar pucker mode simultaneously.

### I-RNA adopts a more extended conformation than A-RNA

We next wanted to compare the overall structures of A-RNA and I-RNA and performed structure calculations of the two dsRNAs based on our NMR data. Even though the dynamic equilibrium of C2′ endo and C3′ endo sugar puckers suggests a more complex picture, we wished to explore a hypothetical I-RNA structure where all sugar puckers in the central region would adopt a pure C2′ endo conformation. Thus, all nucleotides in the hyper-edited motif with ^3^*J*(H1′,H2′) > 3 Hz were restrained to a C2′ endo conformation, while the residues with slightly elevated ^3^*J*(H1′,H2′) (< 3 Hz) were left unrestrained. The regions flanking the central motif were assumed to adopt A-form geometry, which is due to the following observations: Firstly, the imino signals corresponding to these regions are very similar in ^1^H, ^15^N-sfHMQC spectra of I-RNA and A-RNA (Supplementary Fig. S6B). In addition, CD spectra of A- and I-RNA both show the characteristic pattern of A-form RNA, with maxima at 265 nm and minima around 240 nm (Supplementary Fig. S6C). For I-RNA, slight changes in the position and height of maxima indicate a local conformational effect due to the incorporation of inosine residues, which does not affect the flanking regions. This is supported by the imino and aromatic–anomeric walks observed in NOESY spectra for these regions. The backbone torsional angles for the central region of I-RNA were restrained in a loose A-form conformation since, despite conformational changes in this region, the aromatic–anomeric walk is seen in both strands, suggesting that this motif retains some degree of A-form helicity (Supplementary Fig. S6A). In the NOE spectra of I-RNA, all the observed H8/H6-H1’ NOEs are weak, so that all bases are in the *anti*-conformation and were restrained accordingly. A structural model of I-RNA was calculated based on NOE and torsion angle restraints and compared with an NOE-derived structure of A-RNA (Table 1). Comparison of these two RNA duplexes shows that I-RNA adopts a more extended structure due to the presence of four consecutive base pairs with C2′ endo sugar puckers (Fig. 3D).

We performed SEC-SAXS measurements of I-RNA and A-RNA to assess the overall shape of the two RNAs in solution. Frames from the chromatograms with constant radius of gyration (*R*_g_) were selected, and the molecular weight was back-calculated using a Guinier plot (Supplementary Fig. S9). The back-calculated molecular weights agree well with the theoretical values, indicating that both dsRNAs are monomeric duplexes. When comparing the pairwise distance distribution P(*r*) derived from the SAXS data for both RNAs, we find a larger maximum pairwise distance (*D*_max_) for I-RNA (6.89 nm) compared to A-RNA (6.24 nm), indicating a more extended conformation for I-RNA (Fig. 3E). This is also consistent with a slightly increased radius of gyration for I-RNA (*R*_g_ = 1.97 nm) compared to A-RNA (1.83 nm). Finally, the pairwise distance distribution of I-RNA shows a second maximum, which is consistent with a shape comprising two domains connected with a linker, likely corresponding to the dynamic central motif. We then used the SAXS data to validate the NMR structural models. Thus, whereas the A-RNA structure shows good agreement with the SAXS data (*χ*^2^= 1.28), the hypothetical I-RNA model does not agree with the experimental SAXS data (*χ*^2^= 11.93) (Fig. 3F). The residuals show systematic deviations in the lower *q* values, which correspond to larger interatomic distances. This indicates that the more extended conformation, which reflects the C2′ endo conformation in the central hyper-edited motif, does not represent a major populated structure of the I-RNA, consistent with the smaller *J*-coupling constants, which indicate only a fractional population of C2′-endo puckers. The disagreement of the I-RNA structural model and the experimental SAXS data confirms that I-RNA cannot be represented by a single conformation but adopts multiple conformations with dynamically averaged C2′/C3′ endo sugar puckers in the central region.

### The hyper-edited I-RNA adopts a dynamic ensemble of conformations

**Figure 4.**
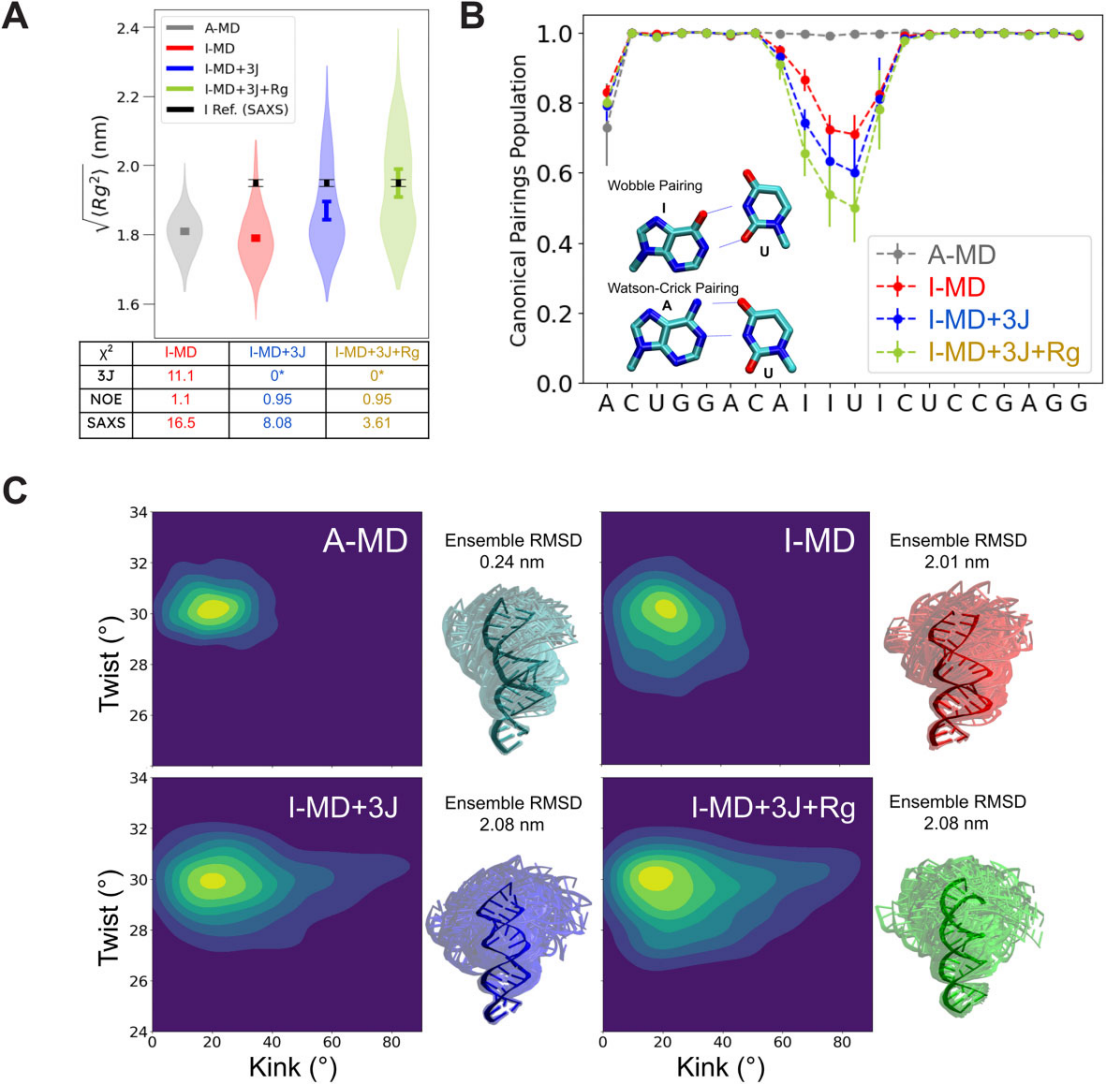
(**A**) Distribution of radius of gyration values of different MD-generated ensembles. A-RNA (gray), I-RNA (red) (both using a RECT sampling scheme), I-RNA with RECT/ME sampling scheme using ^3^*J*(H1′,H2′) values (blue), and I-RNA with RECT/ME sampling scheme using ^3^*J*(H1′,H2′) values and *R*_g_^2^ (green). The *χ*^2^ values in respect to the ^3^*J* couplings, NOEs and full SAXS spectra are listed. (**B**) Populations of canonical pairings of the different ensembles shown in panel (A). (**C**) Plots of twist angles against kink angles of the different ensembles shown in panel (A) and *bouquet* representations with respective RMSD values. The *bouquets* were generated with 100 structures randomly extracted from each ensemble and aligned with respect to the eight residues at the bottom of the helix. The centroids of the ensembles are opaque colored. The ensemble RMSDs are computed on the whole ensembles with respect to the same alignment of the *bouquets*, using the centroids as a reference.

**Figure 5.**
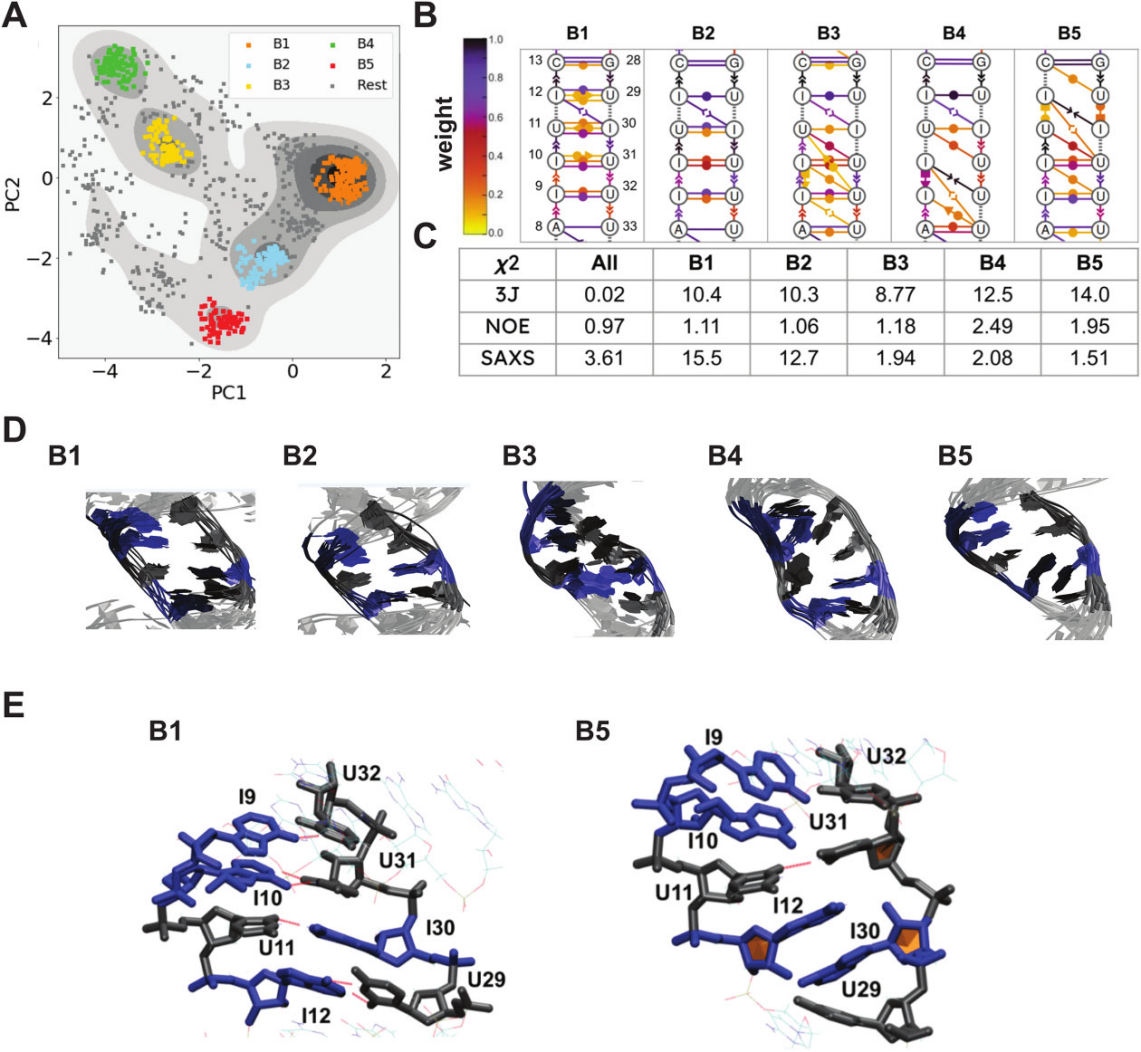
(**A**) 2D density plot of a principal components analysis using torsional angles and G vectors as input. A centroid-based clustering analysis was performed. (**B**) Dynamic secondary structures of the five clusters shown in panel (B) using the Leontis–Westhof notation, the population of the interactions is color-coded accordingly. (**C**) The *χ*^2^ values for the agreement of the individual clusters for ^3^*J*, NOEs, and full SAXS spectra are shown for the individual clusters (B1–B5) and the combination of the clusters (“All”). (**D**) Individual structures of the five clusters (B1–B5) are overlaid in respect to the central motif. Inosines are shown in blue, and uracils are shown in black. The flanking regions are shown in light gray. (**E**) Conformational representative for clusters B1 and B5. A non-canonical stacking interaction between residues I12 and I30 is observed in B5.

In order to obtain a quantitative picture of the conformational dynamics of I-RNA, we generated ensembles using MD simulations in combination with experimental data. We tested different MD-generated ensembles for I-RNA (Fig. 4A): as predicted by MD alone (I-MD), reweighted through ME to enforce ^3^*J*(H1′,H2′) couplings (I-MD + ^3^*J*), and reweighted through ME in order to enforce ^3^*J*(H1′,H2′) and *R*_g_^2^ simultaneously (I-MD + ^3^*J* + Rg). Our findings indicate that the MD simulation alone highly underestimates the ribose C2′ endo population of nucleotides in the central motif and fails to predict ensembles that are consistent with the experimental data (Fig. 4A and Supplementary Fig. S17). However, by using ME reweighting [62] (see “Materials and methods” section), we enforce the agreement with the experimental, dynamically averaged ^3^*J*-couplings and averaged *R*_g_^2^, while achieving improved agreement with other experimental data that were not considered in the ensemble refinement process, such as experimental NOEs and the complete SAXS spectra (Fig. 4A and Supplementary Figs S12 and S13). As expected, the distribution of *R*_g_ values of I-RNA is broader than the one of A-RNA for all the I-RNA ensembles (Fig. 4A). The ensemble of I-RNA also shows a higher propensity of base-pair opening in the central motif. Interestingly, the propensities for open states in the central motif show the same asymmetry as seen in the NMR measurements, in which A8:U33 appeared to be less stable than C13:G28. Also, I12:U29 is more stable in the simulation than the other I:U base pairs, which is consistent with the NMR data, which show observable imino resonances as a result of more stable hydrogen bonding (Fig. 4B). When further analyzing the conformational ensemble, the I-RNA molecular dynamics (I-RNA MD) ensemble shows a greater conformational heterogeneity, specifically adopting conformations with smaller twist angles (averages: 30.3° for A-RNA MD and 29.4° for I-RNA MD), indicating partial unwinding of helical regions. When the experimental average is enforced with ME-reweighting, the conformations also appear to be more bent than in the A-RNA ensemble (Fig. 4C). An increased conformational heterogeneity is also seen by the increased ensemble RMSD of 20.8 Å for the I-RNA ensemble compared to 2.4 Å for the A-RNA ensemble. Fitting the ensemble average against the SAXS data in CRYSOL yields a better agreement than for the I-RNA structural model (*χ*^2^ 3.61 versus 11.93) (Fig. 5C).

### The I-RNA ensemble shows multiple distinct conformational species

To further investigate specific conformational states in the ensemble, we performed a principal component analysis (PCA) based on 1000 structures from each of the ensembles (4000 structures in total), using the torsional angles and G-vectors [70] as input. For the ensembles of A-RNA and I-RNA without the ME-reweighting, a homogenous distribution with a single cluster is observed. For I-RNA, this cluster is more spread out over the principal component coordinate space. In both cases, no significant increase in the population of C2′ endo sugar puckers is observed (Supplementary Figs S10 and S12). The ME-reweighted ensembles of I-RNA (I-RNA MD + ^3^*J* and I-RNA MD + ^3^*J*+ *R*_g_, Supplementary Fig. S10) show multiple clusters that sample a wide region in the PCA density plot. For I-RNA MD + ^3^*J*+ *R*_g_ ensemble, five main clusters were identified by the quality threshold algorithm (Fig. 5A) (relative populations in parentheses): B1 (17.3%), B2 (10.2%), B3 (9.6%), B4 (9.3%), and B5 (7.1%). Clusters B1 and B2 correspond to the main cluster observed in the A-RNA simulation and show A-form helicity with C3′ endo sugar puckers and canonical base pairings; also, their average *R*_g_ values correspond to those experimentally determined for A-RNA (18.3 Å, Fig. 3E). Clusters B3, B4, and B5 show a higher average *R*_g_ of 19.50, 20.57, and 20.07 Å, respectively, likely reflecting an increased helical diameter (Supplementary Fig. S13). The conformers of the clusters were also found to occupy distinct regions in the kink/twist plot, indicating distinct conformational features (Supplementary Fig. S14). These clusters show varying numbers of C2′ endo sugar puckers as well as non-canonical base pairing, which leads to more distorted conformations (Fig. 5B). However, when analyzing the most populated combinations of C2′ endo sugar puckers in the central region, we found that only some of the nucleotides adopt a C2′ endo conformation (Supplementary Fig. S11). Notably, cluster B5 is characterized by the disruption of the canonical pairings for base pairs U11:I30, I12:U29, and C13:G28 by switching the stacking order of I12 and I30 (Fig. 5D and E). For base pairs I9:U32 and I10:U31, where the inosines are neighboring in the same strand, base pairings appear more canonical. For I12:U29, the weights of the hydrogen bond pairing are higher than the weights for the other I:U base pairs in clusters B1–B4, which agrees with our NMR data (Fig. 5B). As the overall fit of the SAXS data showed a better agreement than the NMR model, we fitted the single clusters to the SAXS data using CRYSOL (Fig. 5C). Whereas clusters B1 and B2 alone, which largely reflect canonical A-form geometry, do not agree well with the data (*χ*^2^> 10), the clusters with non-canonical geometries (B3, B4, and B5) agree better with the SAXS data (*χ*^2^ 1.94, 2.08, and 1.51) (Supplementary Fig. S15 and S16). Based on the residual plot (Δ/σ, Supplementary Fig. S15), the disagreement is mainly found for lower scattering vectors, which represent larger interatomic distances. However, the *χ*^2^ values for the experimental NOE data are lower for clusters B1 and B2 than for clusters B3, B4, and B5, and further improved when combining all the clusters (0.97). For the *J*-couplings reporting on the sugar puckers, none of the individual clusters shows good agreement with the data, while the complete ensemble shows good agreement with the experimental data, indicated by a good *χ*^2^ value (0.02) (Fig. 5C). Altogether, this underlines the fact that the entire ensemble with multiple conformational states is necessary to explain the experimental data.

### Hyper-edited dsRNA binds with enhanced affinity to Endonuclease V

**Figure 6.**
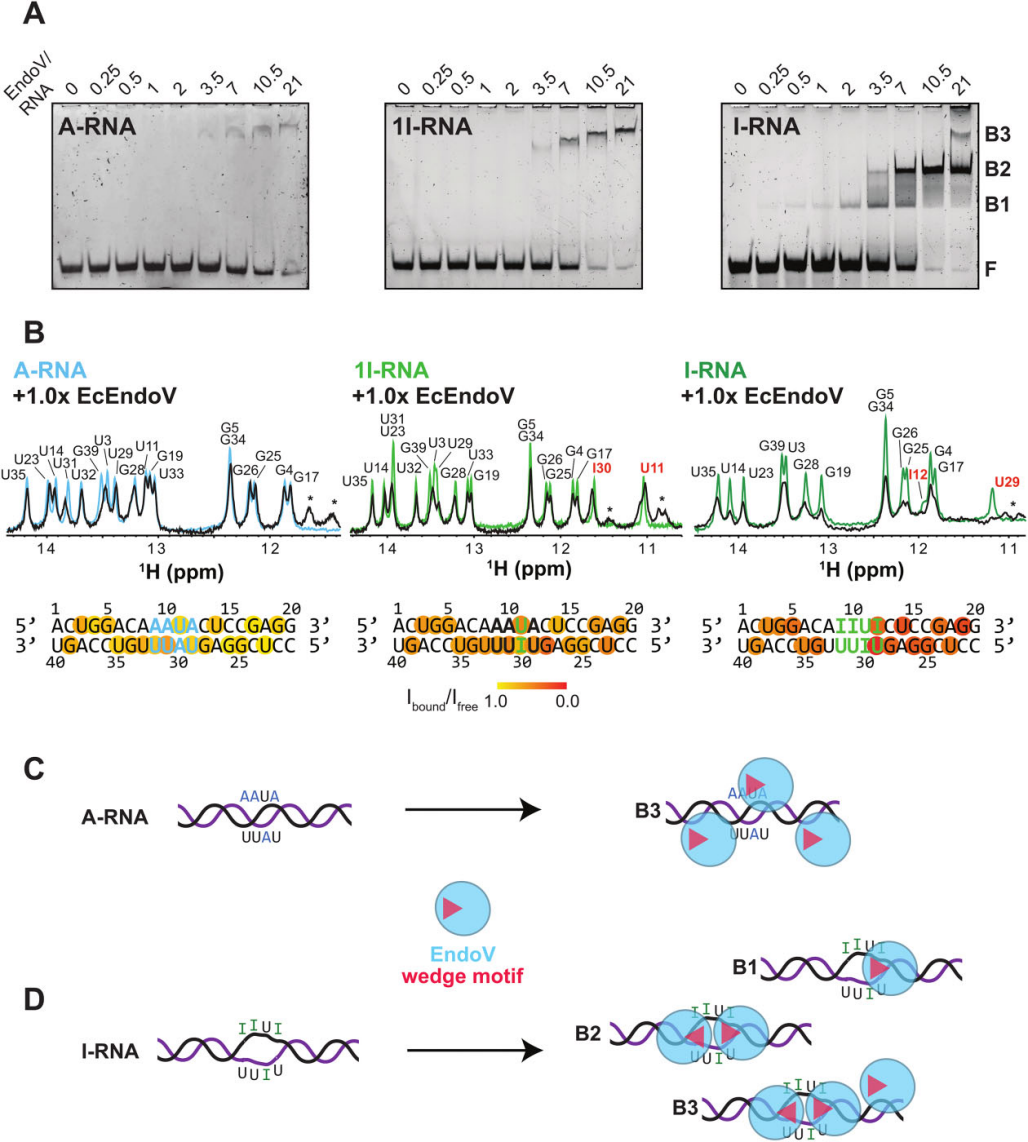
(**A**) Electromobility binding shift assays of *E. coli* Endonuclease V (EndoV) with A-RNA, 1I-RNA, and I-RNA. 0.2 mM RNA was used in each lane. The free and bound RNA forms were separated on 12% native polyacrylamide gels. The free RNA is labeled with "F", bands of protein/RNA complexes are "B1", "B2", and "B3". The gels were stained using SYBR Gold. (**B**) Effect of EndoV binding on the imino resonances of the dsRNAs. Samples containing 50 μM of A-, 1I-, or I-RNA in 25 mM sodium phosphate pH 6.5, 150 mM NaCl, 5 mM MgCl2, 1 mM DTT, and 5% D_2_O were measured at 950 MHz, 288 K as a reference. EcEndoV was added in a molar ratio of 1:1. Asterisks mark protein side chain signals. The observed line broadening was plotted on the sequences. (**C**) Binding of EndoV to A-RNA occurs as non-specific electrostatic interactions. (**D**) Binding of EndoV to I-RNA has different binding modes, with two specific binding modes, B1 and B2, where binding occurs to the hyper-edited central motif as well as an unspecific binding mode, B3, which is similar to A-RNA and 1I-RNA.

To test if conformational features induced upon A-to-I editing are specifically sensed by RNA-binding proteins, we analyzed the interaction with wild-type *E. coli* Endonuclease V (EcEndoV) (residues 1-223), which has been described to show a high specificity in binding to inosine-containing RNAs. We performed EMSAs using the three dsRNAs used in this study. Under the buffer conditions used, in particular a low pH condition, the enzymatic activity of EcEndoV is inhibited (Supplementary Fig. S17A). For A-RNA, a single complex band (B3) is observed at high protein/RNA ratios (Fig. 6A). The fact that the complex band is migrating a shorter distance into the gel at further increased protein concentrations could indicate a non-specific interaction with higher stoichiometries. In the EMSA of 1I-RNA, a similar pattern is observed. As our data indicate that 1I-RNA adopts an A-form helical conformation similar to A-RNA, the interaction observed most likely is the same in both RNAs. This is in agreement with similar trends in line broadening observed for the imino resonances of both dsRNAs upon addition of EcEndoV (Fig. 6B), indicating interactions with the RNA backbone (Fig. 6C). Strikingly, the interactions with I-RNA are completely different (Fig. 6A). The binding affinity is increased, as complex bands are observed at already at low protein/RNA ratios of 0.25:1. The dissociation constant (*K*_D_) of this interaction is estimated to be in the nanomolar range and roughly two-fold lower than the *K*_D_ for A-RNA and 1I-RNA (based on the half saturation of the binding). Strong line-broadening of the imino resonances of I12 and U29, as well as for the adjacent residues G28 and U14, likely reflects an interaction with the central motif and the flanking duplex region (Fig. 6B). At a ratio of 3.5:1, a second band (B2) is observed in the EMSA, suggesting a 2:1 binding stoichiometry of I-RNA to EcEndoV (Fig. 6D). As the imino resonances of the remaining I:U base pairs are not observed in the NMR spectra, an interaction of EcEndoV with the other side of the hyper-edited motif cannot directly be observed. However, pronounced effects on G34 and U35 indicate a stronger interaction of EcEndoV with the flanking duplex region in 5′ of the hyper-edited motif compared to the other RNAs (Fig. 6B). When the protein is added in high excess, a third band (B3) is obtained, which most likely corresponds to the aforementioned non-specific interaction observed for A-RNA and 1I-RNA. When performing EMSAs at increasing salt concentrations, the intensity of this band is decreasing for A-RNA and I-RNA until, at a salt concentration of 500 mM, no shifted band could be observed (Supplementary Fig. S17E). Strikingly, no binding could be detected for A-RNA at this salt concentration, while bands B1 and B2 for I-RNA are still observed, thus showing a highly specific interaction. In general, the binding appears to be dependent on the presence of bivalent cations and can be disrupted by addition of EDTA (Supplementary Fig. S17B). The 2:1 stoichiometry could be explained by binding of EndoV to both strands, as in I-RNA, and the conformational dynamics could render them accessible. However, under the same conditions, no shifted bands were observed for ssRNA substrates with and without inosine, arguing that at least a partial duplex structure is essential for high-affinity binding (Supplementary Fig. S17C and D). The unique binding mode observed for I-RNA is likely due to the unique conformational features of this RNA. The increased base-pair opening as well as undertwisting (Fig. 4) create an extended binding surface, which could allow for binding of EndoV from both ends of the motif, hence explaining the 2:1 stoichiometry (Fig. 6D).

## Discussion

In this study, we investigated the conformation and dynamics of a 20-mer A-to-I hyper-edited dsRNA and assessed its structural and biochemical features in comparison with single- and non-edited versions. This RNA has been studied to assess endonucleolytic cleavage of hyper-edited dsRNA by specifically acting endonucleases [12]. These investigations hypothesized that the pronounced instability of hyper-edited dsRNA is the consequence of an altered RNA structure. Our imino proton NMR data reflect that the incorporation of multiple I:U base pairs indeed strongly destabilizes base-pairing. The extent of this destabilization depends on the number of I:U base pairs and appears to be dependent on the sequence context, as seen from the asymmetric behavior of the central IIUI motif in I-RNA with visible imino resonances for only I12:U29. This likely results from more favorable stacking interactions in this part of the motif, as suggested by varying the orientation of I9:U32, which resembles the same stacking pattern with inosines in opposite strands. Indeed, it is well known that, depending on flanking base types, differential stacking affects the thermodynamic stability of double-stranded helices [71]. Determination of the so-called nearest neighbor effects [71, 72] by optical melting studies on duplexes containing tandem I:U base pairs has revealed that the arrangement with inosines in opposite strands is more stable compared to tandem I:U base pairs with neighboring inosines [19], and structural studies on tandem wobble base pairs have identified more favorable stacking interaction in this configuration [23, 73].

Hydrogen exchange experiments can effectively probe base pair opening in double-stranded nucleic acids [74–76]. We found that the inosine-containing dsRNAs show increased solvent exchange rates, which, in the presence of a single or four consecutive I:U base pairs are increased up to 3-fold and 20-fold, respectively. The *k*_ex_ rates are the net rates of the open-closed equilibrium of the base pair and the exchange from the open state. As this net rate is different from the base-pair opening rate and usually slower, we use the *k*_ex_ values as a semi-quantitative measure of the opening dynamics. In theory, the net rate can approximate the opening rate at increased concentrations of base catalyst so that exchange occurs at every opening event [77]. However, RNA is prone to autohydrolysis at high pH [78], which impairs this type of study. I:U base pairs are thought to adopt similar overall geometries as G:U and A:C^+^ base pairs [23]. The former was described to be more stable than I:U base pairs, and this effect can be assigned to additional stacking interactions of the 2-amino group in guanosine, as due to the lower p*K*_a_ value of the inosine imine (p*K*_a_ = 8.8 compared to 9.2 in G), inosine would be a better hydrogen bond donor [20]. The loss of stabilizing stacking interactions might increase the conformational degrees of freedom in inosine, which then can lead to increased base-pair opening.

So far, only a single crystallographic structure of a palindromic 8-mer dsRNA with tandem I:U base pairs has been reported, which shows an overall canonical A-form helical conformation [23]. Strikingly, the conformation of hyper-edited dsRNAs has not been well studied so far. Based on chemical shift assignments and NOEs seen for anomeric and aromatic positions in our NMR data, the hyper-edited central motif of I-RNA maintains significant levels of a helical conformation. The sugar puckering assessed by ^13^C chemical shifts and ^3^*J*(H1′,H2′) scalar couplings shows a significant propensity for the C2′ endo conformations for nucleotides in the hyper-edited region, which is a hallmark of B-form helices found in dsDNA. Based on the values of the scalar couplings (< 1 Hz for C3′ endo, ∼10 Hz for C2′ endo [38]), we found that none of the residues can exhibit a pure C2′ endo conformation but have a mixed population of C2′ endo and C3′ endo sugar puckers, in which exchange between the conformations might occur on time scales slower than μs–ms.

To provide a structural ensemble representing the dynamical conformations of I-RNA, we combined MD simulations with experimental data, using ME reweighting approach in order to enforce ^3^*J*(H1′,H2′) values and *R*_g_^2^ derived from SAXS (Fig. 4A). In this approach, conformers in an initial ensemble are reweighted to yield a better agreement with experimental data without introducing new conformers. The obtained reweighted ensemble shows excellent agreement with ^3^*J*(H1′,H2′) values *R*_g_^2^ and also yields good agreement with NOEs and full SAXS spectra, indicating the presence of five conformationally distinct clusters. Strikingly, some of the clusters with low populations (B3, B4, B5), in particular, show good agreement with the high q part of the SAXS profile. As only the *R*_g_ has been used as a restraint, this shows that our integrative approach can extract important conformational features that cannot be obtained by conventional MD methods or experiments alone. Structural analysis of the ensemble shows that A-to-I hyper-editing increases the populations of conformations with internal loop-like properties as many canonical base pairs are lost and base-pair opening is increased. However, stacking interactions between the I:U base pairs are still present, such as the non-canonical interaction between I12 and I30. This set of conformational features might create a unique protein-binding surface.

In recent years, few MD studies have addressed inosine modifications. Thermodynamics of I:C pairs has been investigated in RNA and DNA duplexes using alchemical calculations [79], qualitatively reproducing the differences in free energy impact of G to I substitution in B-DNA and A-RNA helices, but somehow underestimating the effect. A more comprehensive study, which included additional sequences and new experimental data, confirmed that MD can accurately predict the differential stability between G:C and I:C pairs [80]. Simulations of duplexes containing single and tandem I:U pairs were performed in [81] but did not report high helical distortions or sugar pucker conformational changes. However, this study considered at most tandem modifications. Interestingly, our I-MD simulations did not suggest significant conformational changes in the hyper-edited region without the explicit integration of experimental information. Note that the force field that we used for the hypoxanthine moiety has been adapted from modrna08, where partial charges were derived assuming the nucleotide to adopt a C3′-endo conformation [54]. This might explain why modrna08 may penalize the C2′-endo conformation. A better parametrization for hypoxanthine might promote the C2′-endo population. On the other hand, an underestimation of C2′-endo population is also observed for the uracils in the central motif, for which partial charges have been derived from both sugar pucker states.

G:U and I:U base pairs exhibit similar base-pairing patterns that would allow for similar conformations in dsRNA. MD studies on both tandem G:U and I:U base pairs revealed complex hydrogen bonding that leads to different base pair geometries, which largely depend on intra- and inter-strand stacking in the motifs [81, 82]. In our simulations, the clusters of more distorted conformations, such as B5, show non-canonical stacking interactions, in particular an interaction between I9 and I30. As such stacking interactions were not observed in either tandem G:U or I:U base pairs, the number of wobble base pairs might be key to introduce conformational distortions in RNA duplexes. While we observed this for A-to-I hyper-edited dsRNA here, the same dsRNA sequence with four G:U wobble base pairs shows only moderate thermal destabilization and a helical conformation as reported in earlier work by our group [26].

To explore the role of the unique conformational features and extensive dynamics for protein recognition of hyper-edited RNA, we studied the interaction with EcEndoV. In fact, we observed increased binding affinity of EcEndoV toward I-RNA compared to A-RNA and 1I-RNA. The complex multistep binding observed is likely linked to the conformational dynamics and heterogeneity of the I-RNA conformational ensemble. In particular, loss of base-pairing leads to internal loop-like properties, which may increase the presence of unwound conformations that could expose inosine in single-stranded conformations. Interestingly, previous studies show that EcEndoV has higher affinity toward ssRNA substrates containing single inosine residues compared to inosine-containing dsRNA, while the effects of multiple I:U base pairs have not been studied so far [16, 83]. A role of hyper-edited dsRNA substrates was investigated in a eukaryotic EndoV homolog [13], where I-RNA has been used as a model system. Our data are consistent with findings from this study, where high-affinity binding for I-RNA and strongly reduced binding to ssRNA were observed. This underscores the importance of a dsRNA context and hyper-editing for EndoV binding. The bacterial homolog EcEndoV has been used in our study as a model inosine RNA-binding protein due to its high binding affinity and specificity [16]. Our binding experiments suggest that the presence of multiple I:U base pairs can be recognized by EndoV as a “lesion”, likely due to its strongly distorted helical geometry. At the same time, duplex features in the adjacent stem regions are maintained, which was shown to be important for EndoV binding in both prokaryotes and eukaryotes [18, 84]. This could act as a mechanism to mask A-form helicity in longer cytosolic dsRNA stretches. As there still is little knowledge about hyper-editing sites in the transcriptome, future work should elucidate the scope of biological functions of A-to-I hyper-editing. To the best of our knowledge, this is the first comprehensive study of the conformation of an A-to-I hyper-edited dsRNA.

## Supplementary Material

gkaf550_Supplemental_File

## Data Availability

The structure of A-RNA and NMR restraint files has been deposited with the PDB (accession code 9I8B). The hypothetical structural model of I-RNA, restraint files, and scripts used for structure calculations are available at Zenodo, https://doi.org/10.5281/zenodo.13886760. Chemical shift assignments and NOE peak lists for A-RNA have been deposited with the BMRB (ID 52888): imino chemical shift assignments for 1I-RNA are deposited at BMRB (ID 52886). Chemical shift assignments, ^3^*J* couplings, and NOE peak lists are available at BMRB (ID 52873). SAXS data of I-RNA and A-RNA have been deposited with SASDB (accession codes SASDWL5 and SASDWM5, respectively). All data and scripts related to molecular dynamics are available at https://doi.org/10.5281/zenodo.13886760.
