# Supplementary material for: Unique conformational dynamics and protein recognition of A-to-I hyper-edited dsRNA
Source: Nucleic Acids Res. 2025 Jun 26;53(12):gkaf550. doi: 10.1093/nar/gkaf550 (PMC12199146; doi:10.1093/nar/gkaf550)
Supplement: gkaf550_Supplemental_File [file gkaf550_supplemental_file.pdf]

Supporting Information

Unique conformational dynamics and protein recognition of A-to-I hyper-edited dsRNA

Christoph Müller-Hermes[1,2], Valerio Piomponi[3], Stefan Hilber[4], Sam Asami[1], Christoph Kreutz[4], Giovanni Bussi[3], Michael Sattler[1,2,5]

[1] Technical University of Munich, TUM School of Natural Sciences, Bavarian NMR Center and Department of Bioscience, 85748 Garching, Germany

[2] Helmholtz Munich, Molecular Targets and Therapeutics Center, Institute of Structural Biology, 85764 Neuherberg, Germany

[3] Scuola Internazionale Superiore di Studi Avanzati, via Bonomea 265, 34136, Trieste, Italy

[4] Institute of Organic Chemistry and Center for Molecular Biosciences, Innsbruck (CMBI), University of Innsbruck, 6020 Innsbruck, Austria

[5] Cluster for Nucleic Acid Therapeutics Munich (CNATM), Munich, Germany)

* To whom correspondence should be addressed. Tel: +49 89 289 52600

E-mail: michael.sattler@helmholtz-munich.de

Contents

Figure S1-S17

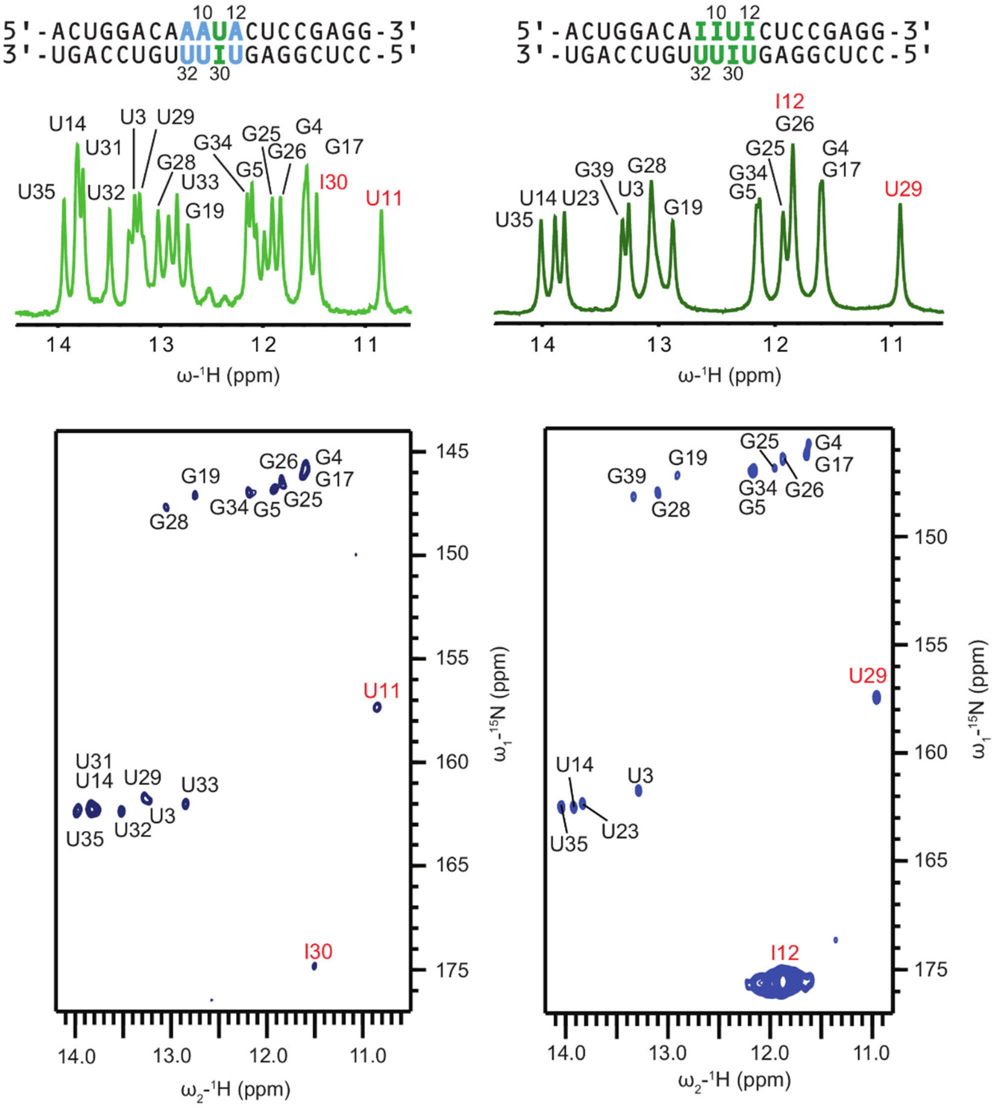

Figure S1: 1D Imino proton and 2D ^{15}N, ^{1}H sfHMQC (at ^{15}N natural abundance) spectra of 1I- and I-RNA. A selectively inosine-^{15}N1-labelled sample was used for the latter. Imino resonances were assigned using ^{1}H,^{1}H NOESY spectra. Imino resonances of I:U base-pairs were observed in the upfield part of the imino region, similar to G:U base-pairs. For the ^{15}N chemical shifts, the inosine N1 shows a characteristic downfield chemical shift at ca. 175 ppm. For I-RNA, only the I:U base-pair I12:U29 is observed.

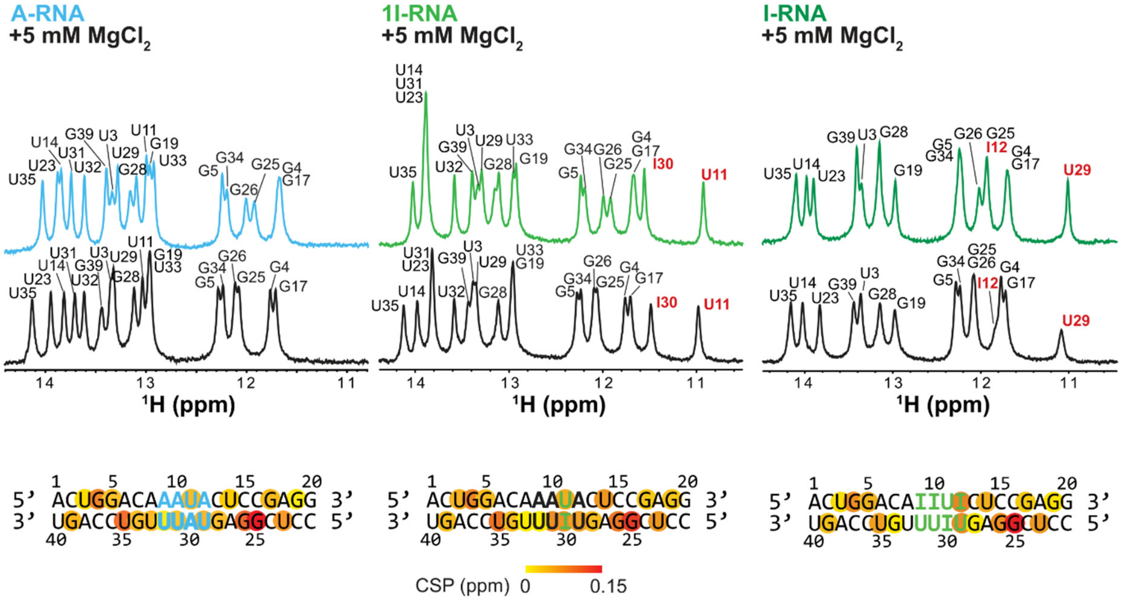

Figure S2. Effect of Mg^{2+} binding on the A-, 1I- and I-RNA. 100 µM samples of the dsRNAs were prepared in 25 mM NaP_i pH 6.5, 25 mM NaCl. $MgCl_2$ was added to a final concentration of 5 mM. Spectra were recorded at 278 K at 950 MHz. The observed chemical shift perturbations (CSP) are plotted on the sequences. The stretches of the I:U base-pairs do not show increased effects upon addition of Mg^{2+}.

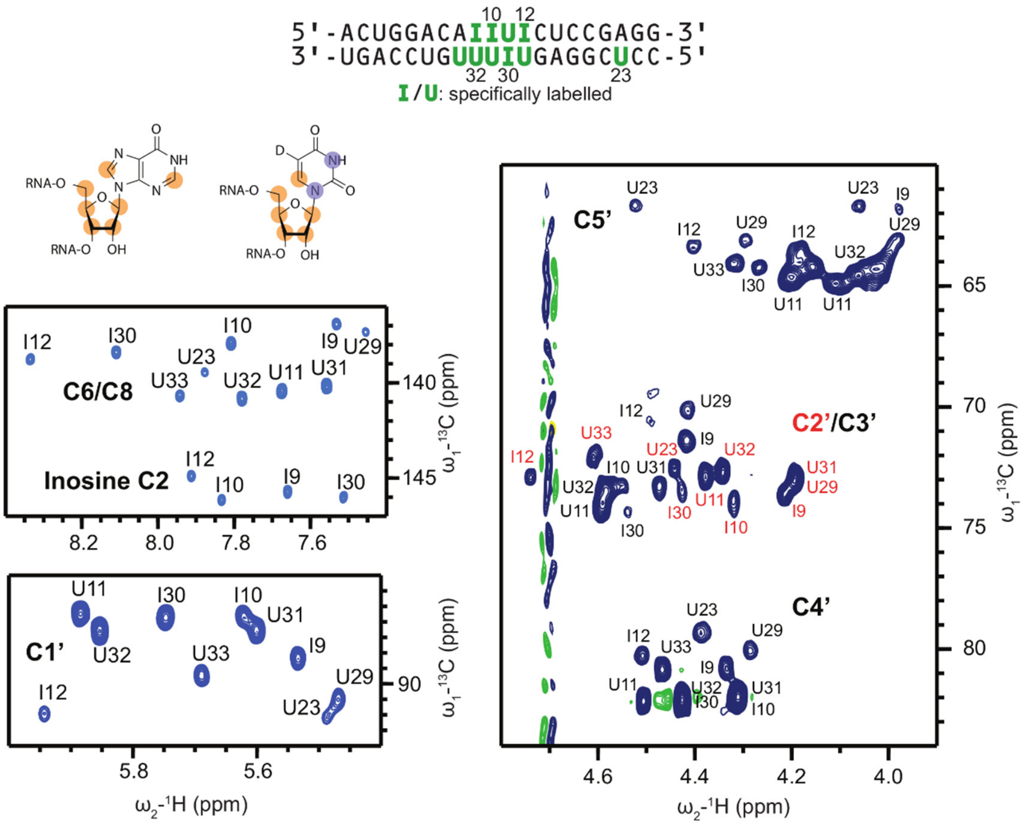

Figure S3: HSQC spectra and assignments of selectively $^{13}C/^{15}N$-labelled I-RNA. The $^{13}C/^{15}N$ labeling pattern is shown. All nucleobase (inosine C8 and C2, uracil U6) and sugar resonances (C1‘, C2‘, C3‘, C4‘, C5‘/C5‘‘) of the selectively labeled residues could be assigned. Assignments were obtained from 3D NOESY-HMQC, HCCH-TOCSY and HCCH-COSY experiments. Uracil H5 were exchanged for deuterium to remove scalar coupling to H6-C6. Besides the central motif and U33, which did not give rise to an observable imino proton signal, U23 was labeled which is found in one of the flanking regions which are expected to show A-form helicity.

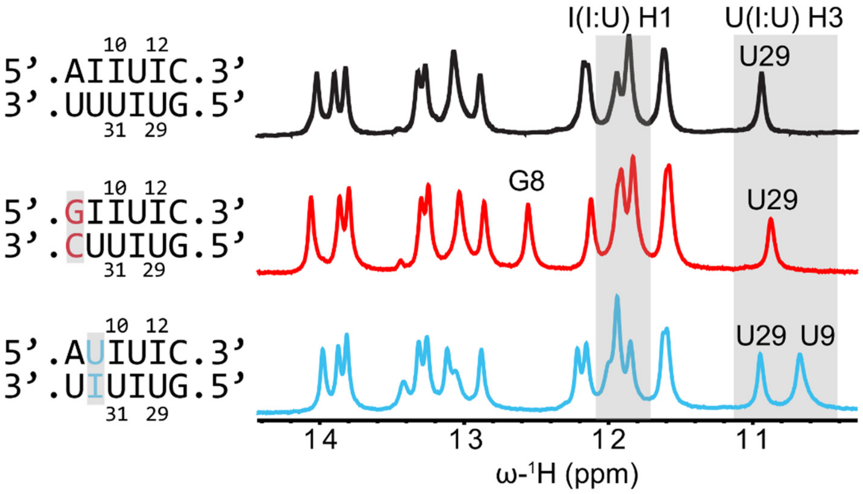

Figure S4: 1D imino proton spectra of I-RNA (black), I-RNA in which A8:U33 was replaced by G8:C33 (red) and I-RNA in which I9:U32 was flipped to U9:I32 (blue). Only for the latter additional imino signals corresponding to I:U base-pairs are observed. This indicates that the asymmetrical behavior of the central motif is not due to stabilizing effects of neighboring base-pairs (A8:U33 and C13:G28), but that the I9:U32/I10:U31 motif seems to have less favorable stacking interactions. As the stacking is more favorable in U9:I32/I10:U31, the base-pairing in U9:I32 gets stabilized, which leads to observable imino proton resonances.

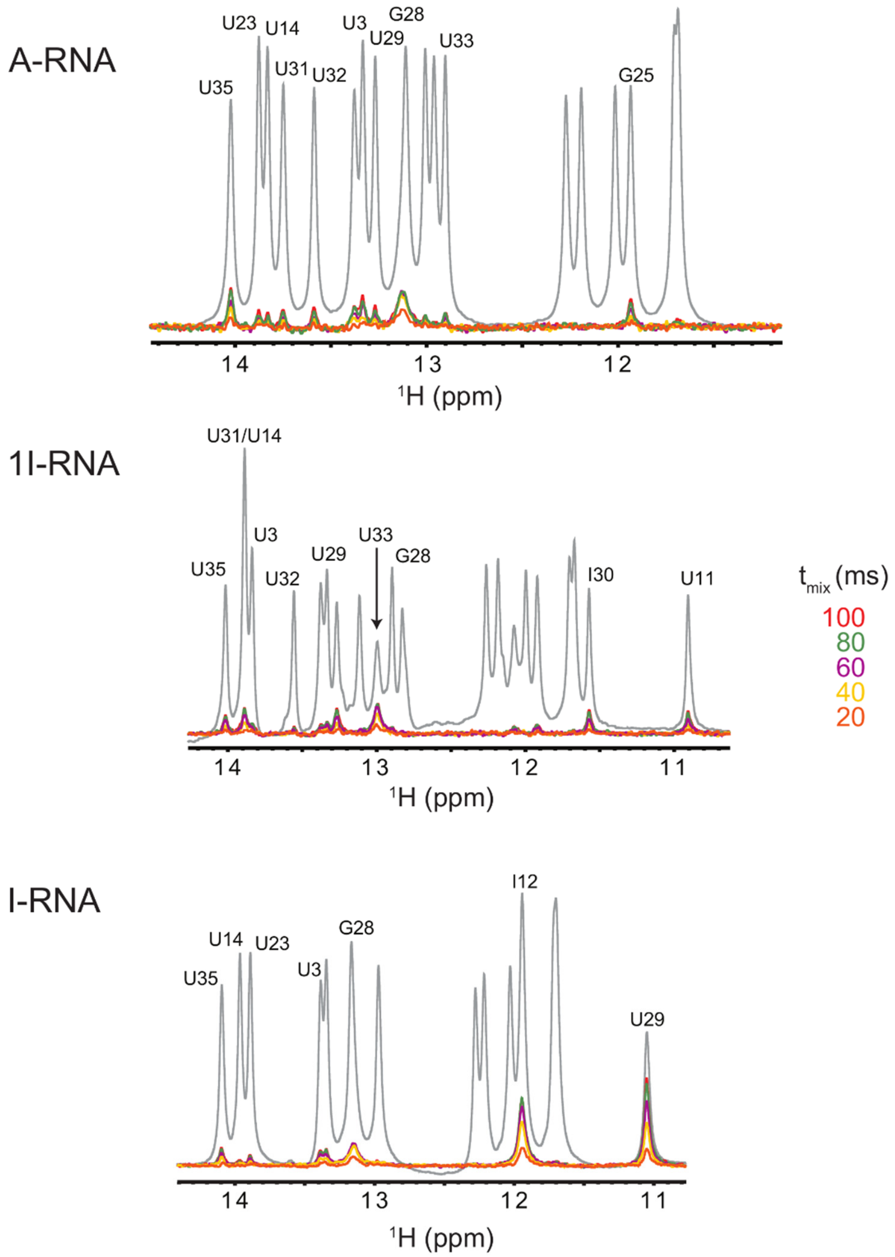

Figure S5: 1D CLEANEX-PM spectra at different mixing times. The spectra at different mixing times are color-coded. In grey, a reference 1D spectrum is shown. Assignments of exchanging signals are indicated. The peak heights were extracted and used for the analysis. I:U base-pairs show intense signals in the CLEANEX-PM spectra, especially in the case of hyper-edited dsRNA (I-RNA), indicating increased solvent exchange on the ms-s time scale.

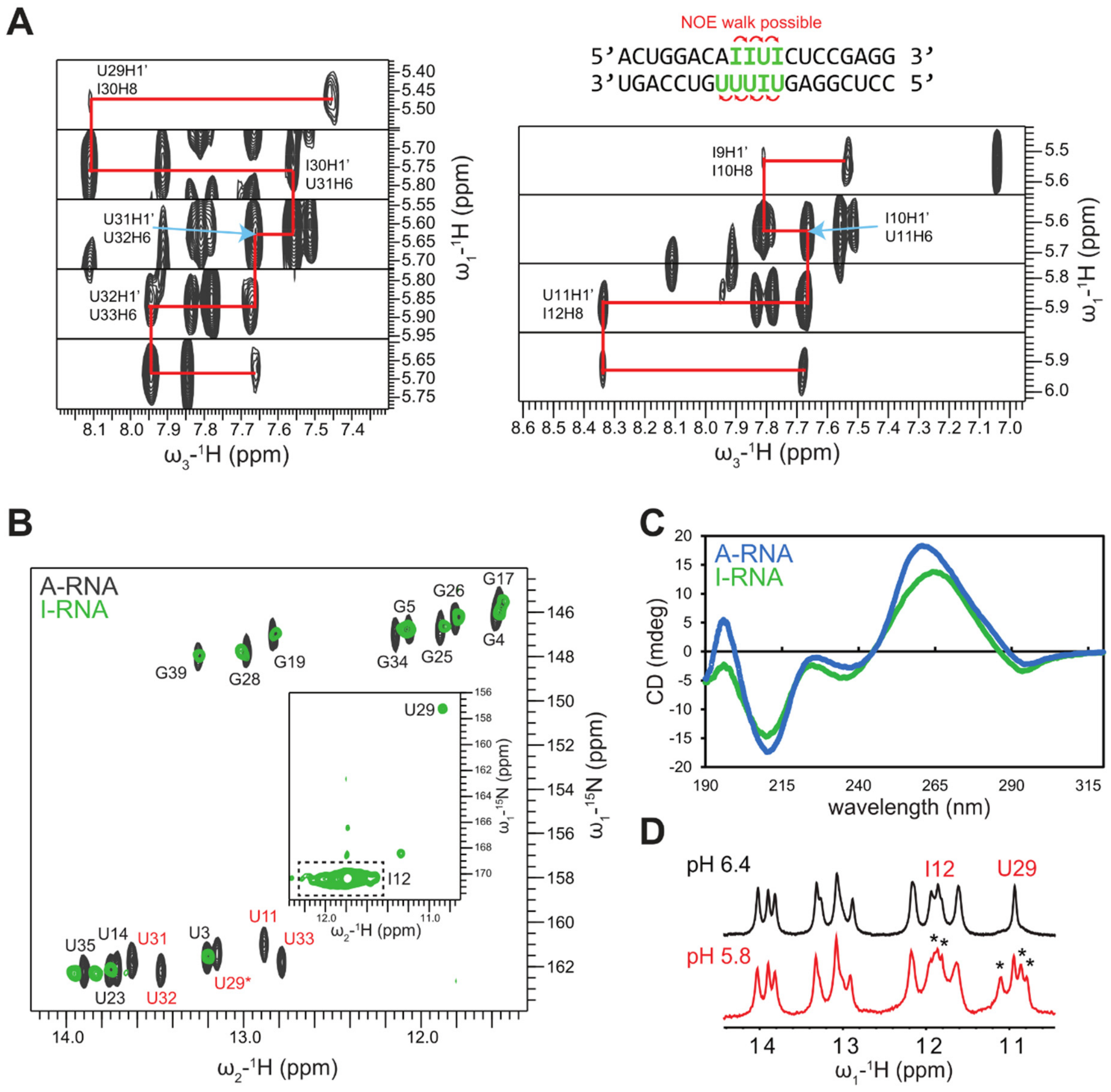

Figure S6: I-RNA shows a high degree of A-form helicity. (A) Strips of a 3D NOESY-HMQC spectrum showing the aromatic-anomeric walks for both strands of the central motif. Assignments of residues of the central motif are indicated. Since the aromatic-anomeric walk is possible, I-RNA shows A-form helical features for the central motif. (B) Overlay of ^{15}N, ^{1}H sfHMQC (at ^{15}N natural abundance) of A-RNA (black) and I-RNA (green). The central motif residues U11, U29, U31, U32 and U33 are marked in red since they, for I-RNA, are not observed or observed at a different frequency (U29). Residues of the flanking regions are in good agreement, indicating a similar conformation. (C) CD spectra of A-RNA (blue) and I-RNA (green). Both spectra show A-form dsRNA features. Slight changes in I-RNA are indicative of local conformational changes. (D) 1D imino proton spectrum of I-RNA at pH 6.4 (black) and pH 5.8 (red). Additional signals at the positions of imino protons of I:U base-pairs are observed at lower pH, indicating that missing imino signals in I-RNA are due to fast solvent exchange.

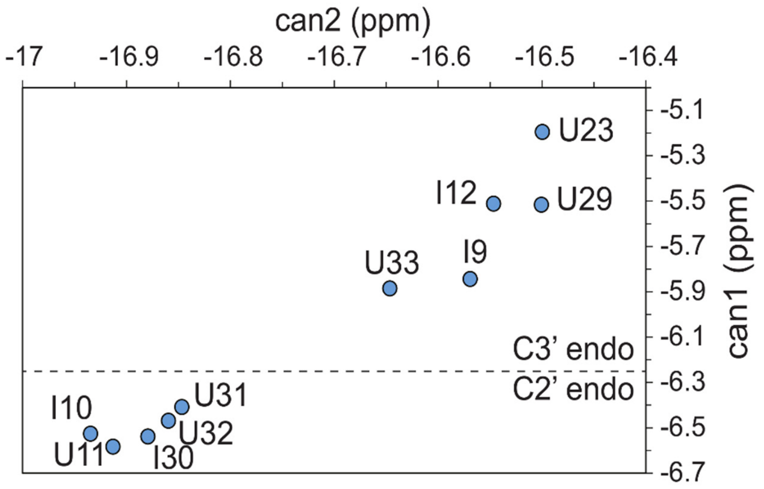

Figure S7: Values of the canonical coordinates can1 against can2, derived from ^{13}C chemical shifts. The central motif residues I10, U11, I30, U31 and U32 show can1 values smaller than -6.25 ppm, indicating that these nucleotides adopt the C2'endo conformation. I9, I12, U23, U29 and U33 adopt the canonical C3'endo conformation. As the can2 values of all nucleotides are greater than -17.2 ppm, it can be assumed that all residues are gauche-gauche about the C5'-C4' bond, which is characteristic for A-form helicity.

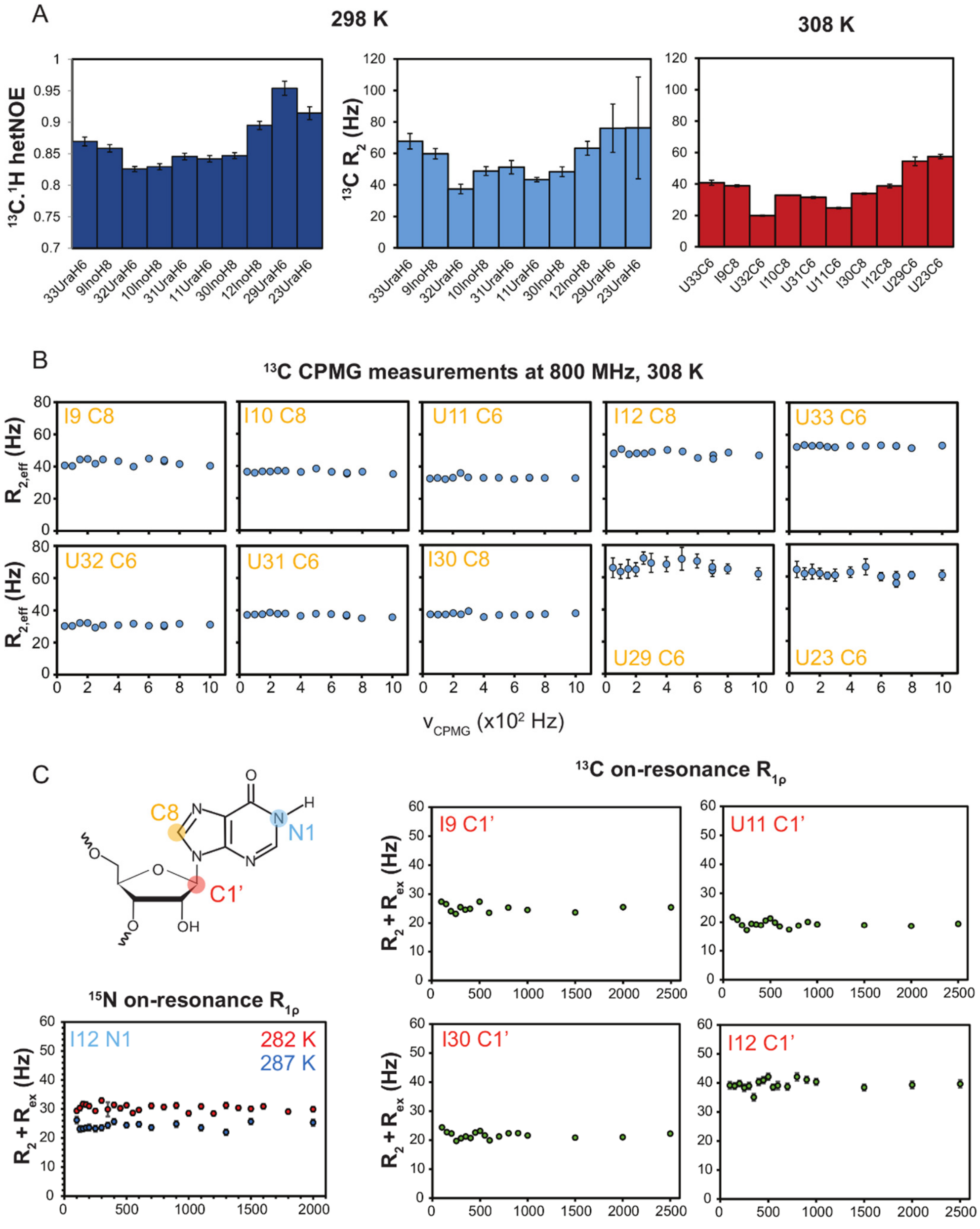

Figure S8: Relaxation experiments to probe dynamics at different time scales, recorded on selectively ^{13}C-labelled I-RNA. (A) Measurements of ^{13}C, ^{1}H hetNOE (at 298 K) and ^{13}C R_2 (at 298 and 308 K) reflect dynamics on the ps-ns time scale. Lower values of these parameters indicate increased dynamics. Residues of the central motif show lower values than residues of the flanking parts (i.e. U23). (B) ^{13}C CPMG relaxation dispersion was recorded for the aromatic signals of selectively ^{13}C-labelled I-RNA. These measurements reflect dynamic processes on the µs-ms time scale, such as flipping out of bases from the duplex. Flat profiles indicate the absence of such dynamics. (C) On-resonance $R_{1\rho}$ relaxation dispersion experiments were recorded for a selectively inosine-^{15}N1-labelled (recorded at 800 MHz for 282 and 287 K) and selectively ^{13}C-labelled I-RNA (recorded at 800 MHz and 308 K). These experiments show µs-ms time scale dynamics which for imino resonances indicate alternative base-pairing, such as transient Hoogsteen base-pairing and sugar puckering dynamics for the anomeric C1‘position. Flat relaxation dispersion profiles indicate the absence of such dynamics in I-RNA.

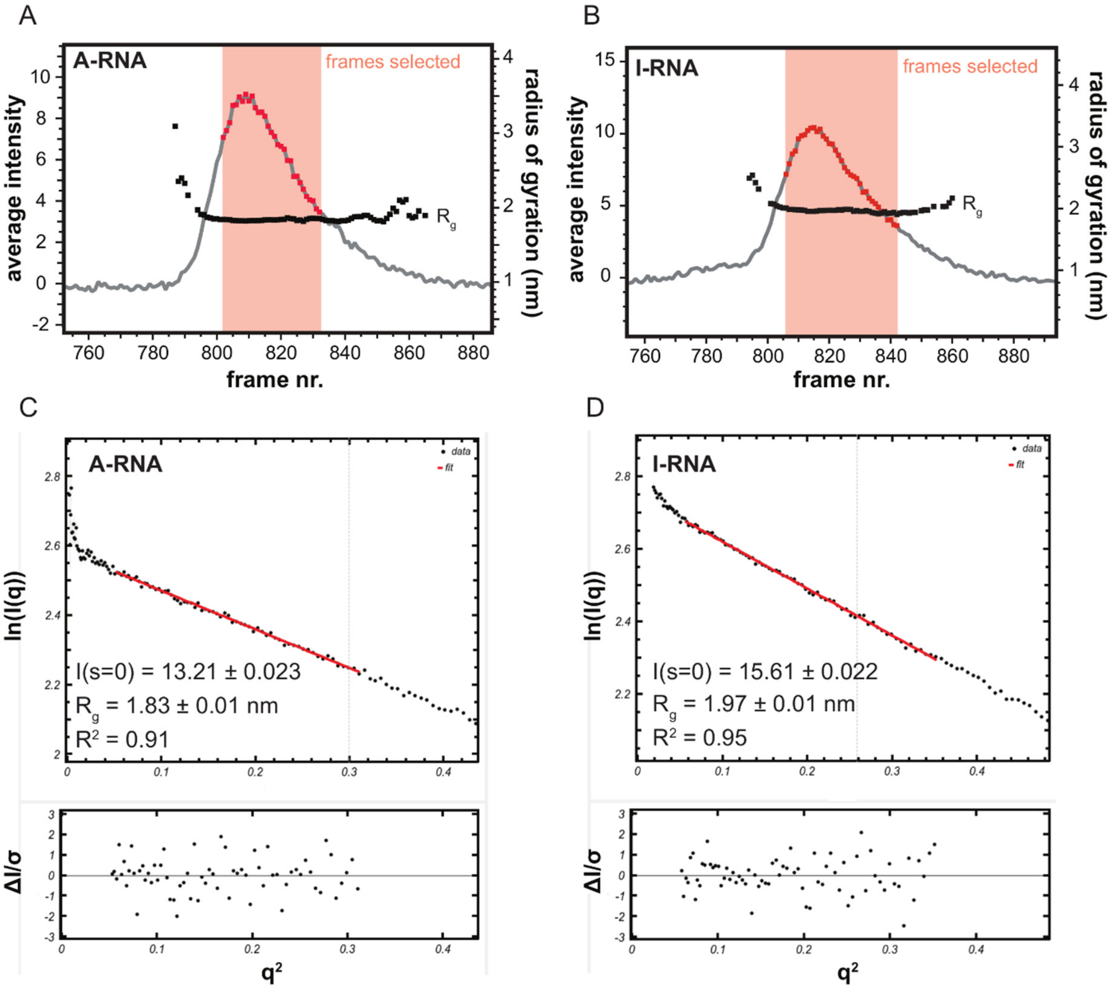

	A-RNA	I-RNA
**backcalculated MW (Da)**	12866	13507
**theoretical MW (Da)**	12734	12739

Figure S9: SEC-SAXS chromatograms of A-RNA (A) and I-RNA (B). Frames with constant R_g value were selected and a buffer subtraction was performed in CHROMIXS. Data was processed analyzed in PRIMUS. Guinier plots of A-RNA (C) and I-RNA (D) were analysed in order to determine the R_g. Points in the initial slope of the curve were selected to yield a $R^2 \geq 0.9$. The initial non-linear part was avoided, which is a consequence of the interstrand interactions of the duplexes. Molecular weights were back-calculated and compared with the theoretical molecular weights as validation. The back-calculated MW values indicate that both dsRNAs are monomeric.

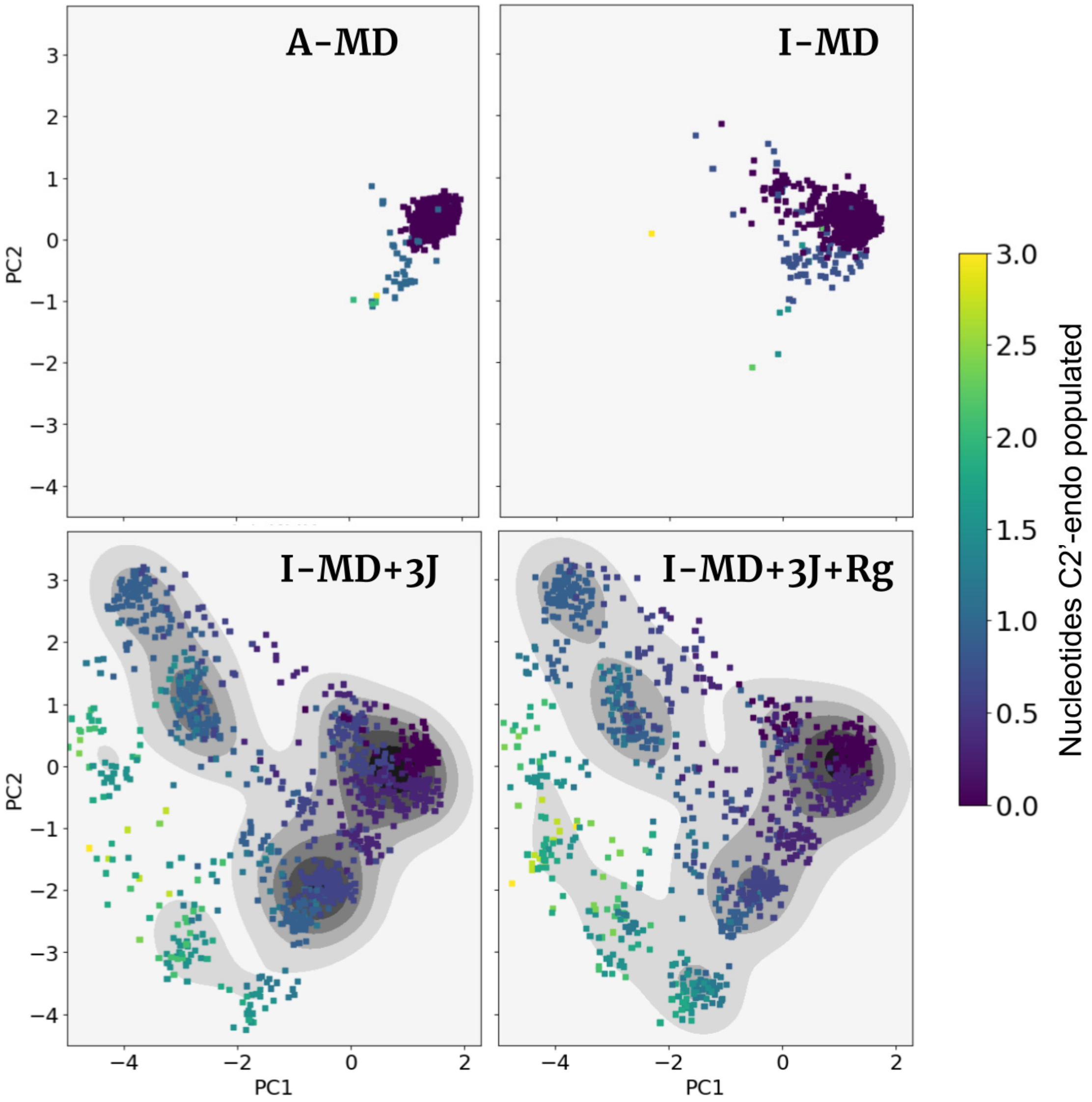

Figure S10: Results of principal component analysis (PCA) performed using backbone torsional angles and G-vectors for the eight central nucleotides from 4000 structures (1000 structures per ensemble) as input. For each ensemble, a 2D density plot with respect to the first and second principal component is shown. The color-coding of the dots indicates the number of nucleotides in C2'-endo conformation.

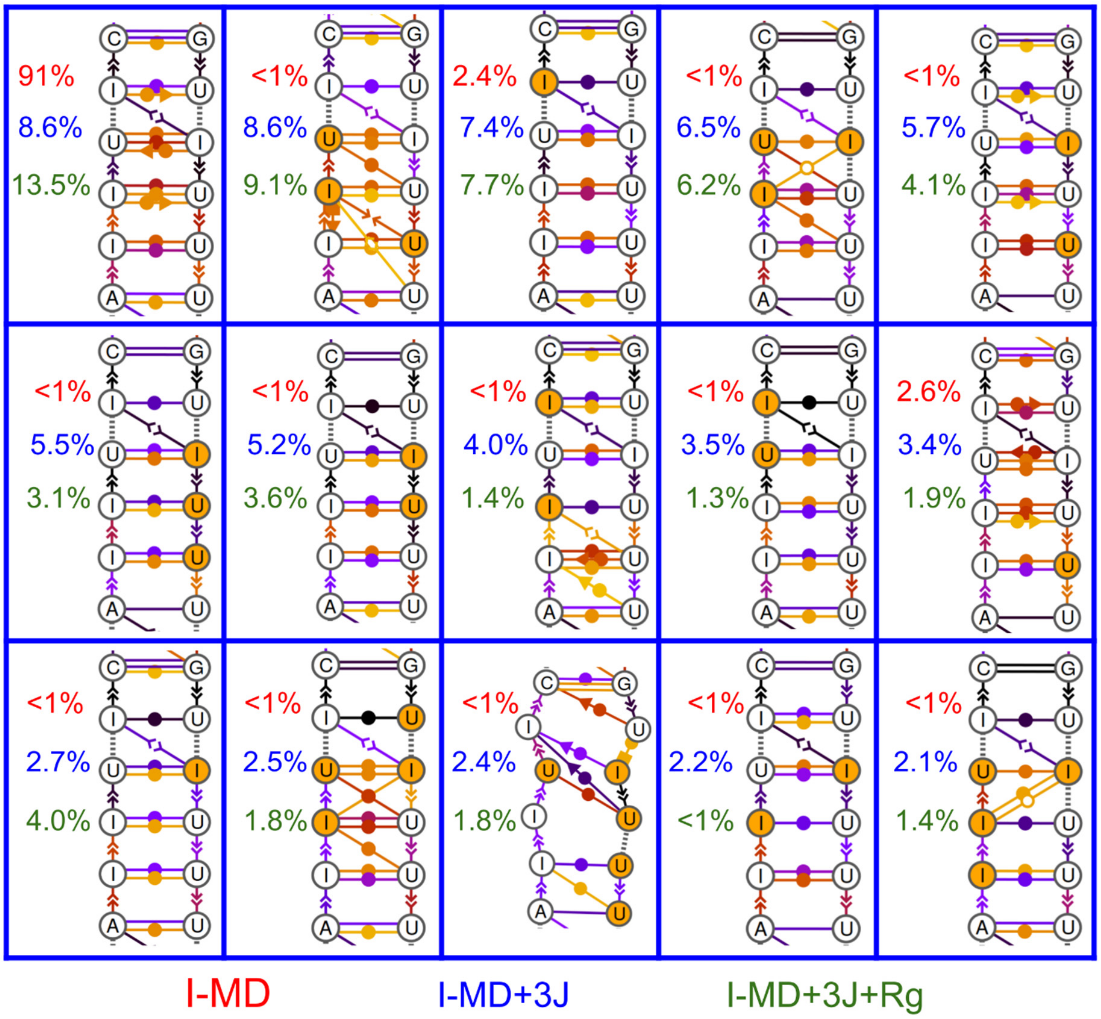

Figure S11: Dynamic secondary structures of 15 conformers corresponding to different possible combinations of sugars in C2'-endo conformations, which are colored in orange. Populations for these 15 cases are shown for the I-MD ensemble (red), the I-MD+3J ensemble (blue), and the I-MD+3J+Rg ensemble (green). The dsRNAs are shown only for the 12 central nucleotides using the dynamic secondary structure representation derived using pdbs extracted from the I-MD+3J ensemble.

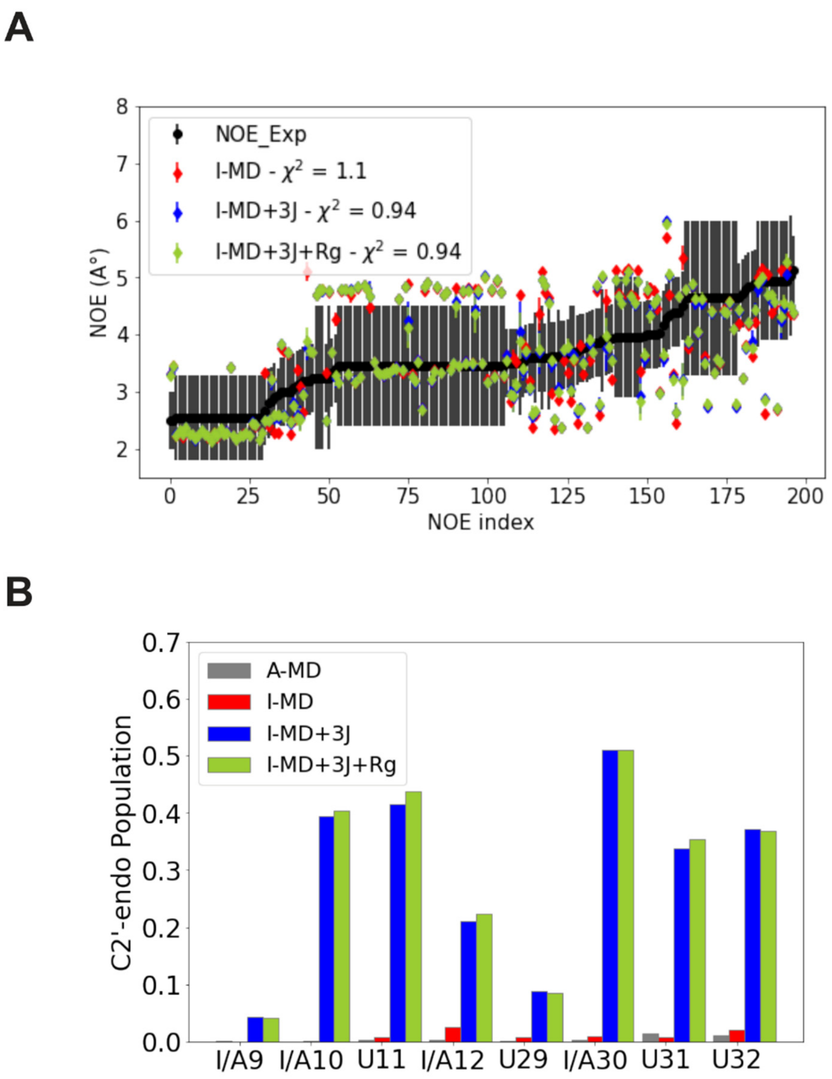

Figure S12: Plot of experimental NOE values (black), ordered in respect to the mean values. Back-calculated NOEs from the trajectories of the different ensembles (I-MD: red; I-MD+3J: blue; I-MD+3J+Rg: green). For each of the three ensembles, the averaged χ^2 value is shown in the legend. Populations of ribose C2'-endo conformations for the central nucleotides for the four different MD generated ensembles. The populations increase significantly, by orders of magnitude, when incorporating NMR data. This increase is a consequence of the inaccuracies of molecular dynamics (MD) simulations using the currently available force-field, which results in a substantial underestimation of these populations for this system. However, by combining enhanced sampling and ensemble refinement techniques, we can accurately reproduce the populations that align with the predictions from the ^{3}J scalar couplings. These results underscore the need for a revision of the inosine force-field, and possibly also for the other nucleotide force-fields, since also the C2'-endo populations of uracils in the central motif are highly underestimated in the I-MD (red) ensemble.

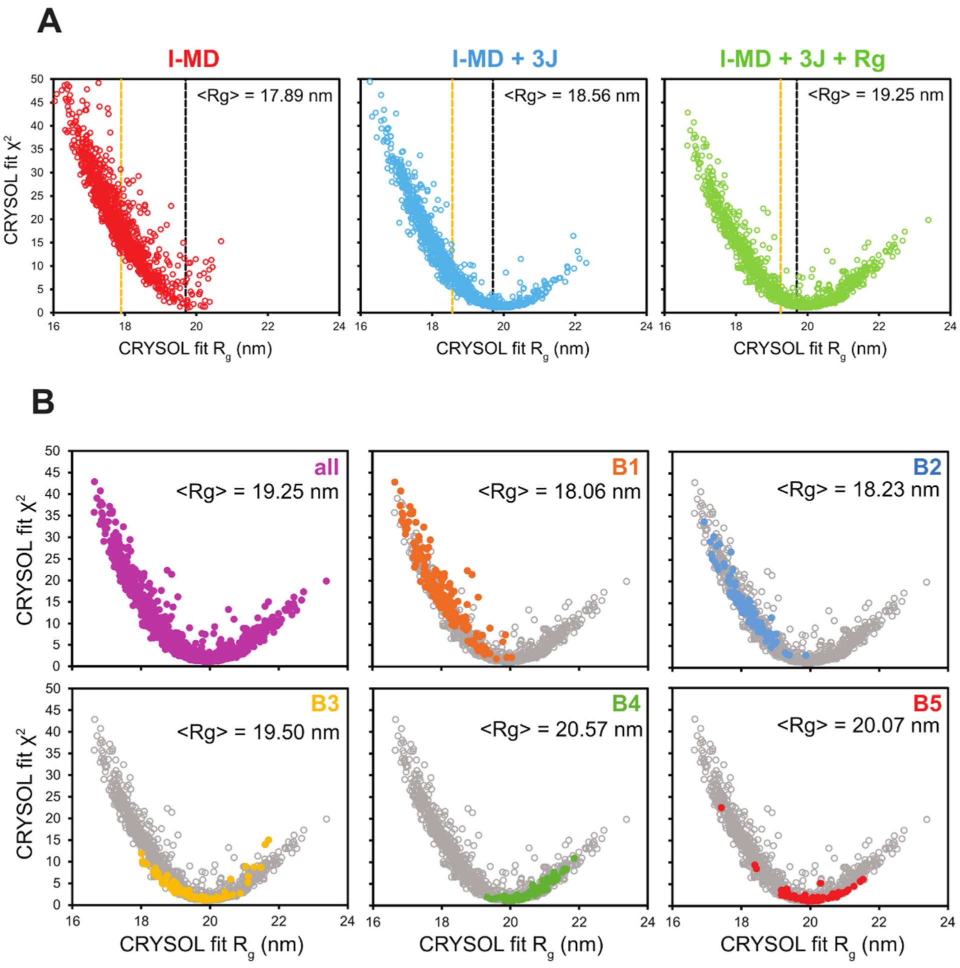

Figure S13: (A) Plots of the CRYSOL fit R_g against the CRYSOL fit χ^2 for 1000 snapshots (open circles) of the three ensembles “I-MD” (red; no experimental restraints), “I+3J” (blue; ^{3}J-coupling restraints) and “I+3J+Rg” (green; ^{3}J coupling and R_g^2 restraints). The black dashed line indicates the experimentally determined R_g for I-RNA (19.7 Å). The yellow dashed line shows the average R_g of the respective ensembles which is also shown in the plots. The use of maximum entropy reweighting and additional experimental data improves the agreement of the ensembles with the SAXS data. (B) Plots of the CRYSOL fit R_g against the CRYSOL fit χ^2 for the individual clusters identified by principal component analysis for the “I+3J+Rg” ensemble. The distribution for all 1000 snapshots is shown as “all“ and as grey open circles. The clusters adopt distinct parts of the distribution, indicating that they are conformationally distinct. Clusters B1 and B2 are found in the region that was obtained for the “I-MD” ensemble (Fig. S9), whereas clusters B3, B4 and B5 show more non-canonical conformations.

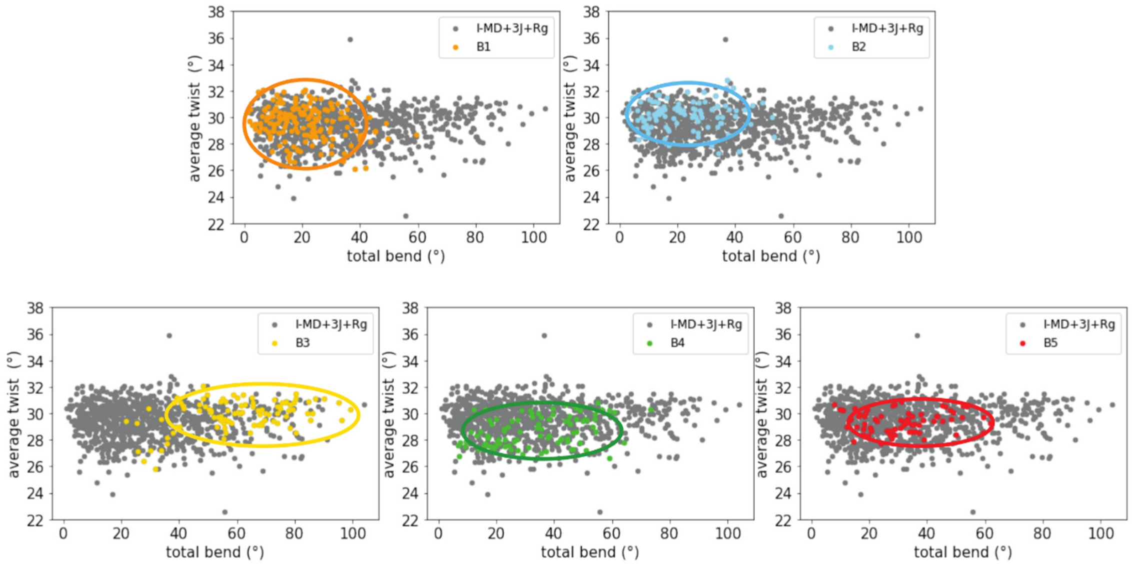

Figure S14: Plot of average twist against total bend for the snapshots of the I+NMR+SAXS ensemble (grey dots). Clusters B1, B2, B3, B4 and B5 are indicated as colored dots, ellipses show the outline of the clusters. Since the individual clusters correspond to distinct regions of the plot, they appear to adopt distinct conformations.

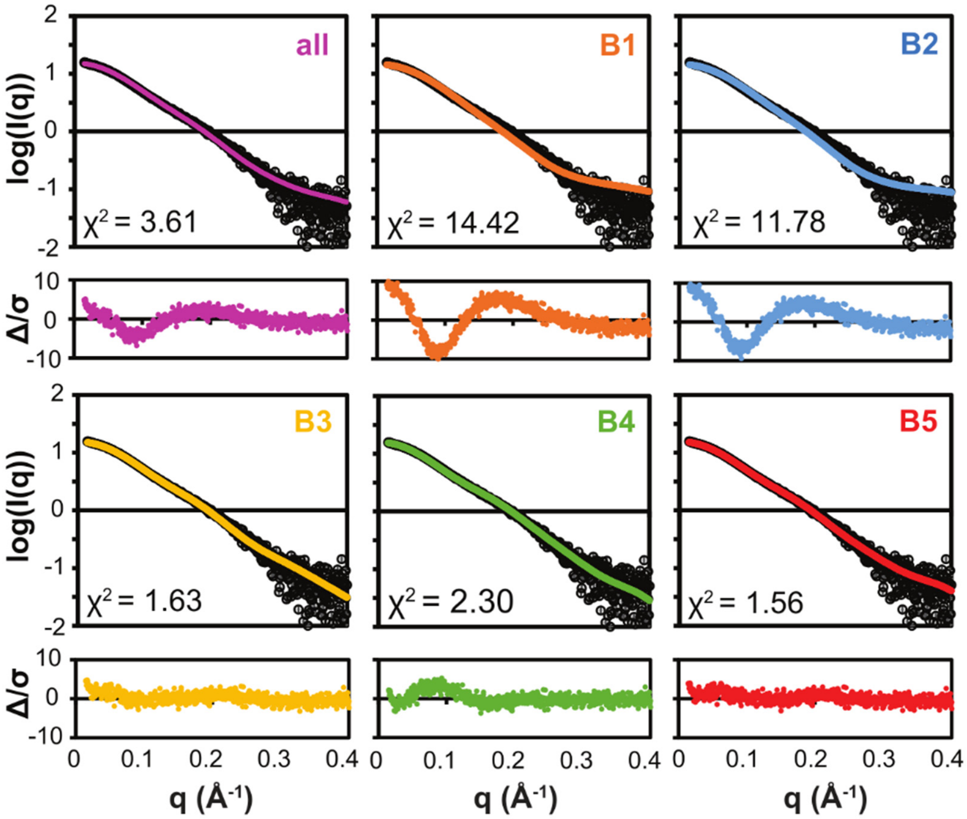

Figure S15: CRYSOL fits of the 1000 snapshots of the I+NMR+SAXS-ensemble and for the individual clusters. Experimental SAXS curves are shown as black open circles, fit curves are shown as a line. The fit residuals Δ/σ are shown underneath the fits. The entire ensemble ("all"; purple) shows a better agreement with the SAXS data than the fit of the NMR model (χ^2 = 11.93). Whereas clusters B1 and B2, which are representing more A-form-like conformation, show worse agreement with SAXS data, clusters B3, B4 and B5 which contain more non-canonical conformations show better agreement with the SAXS data.

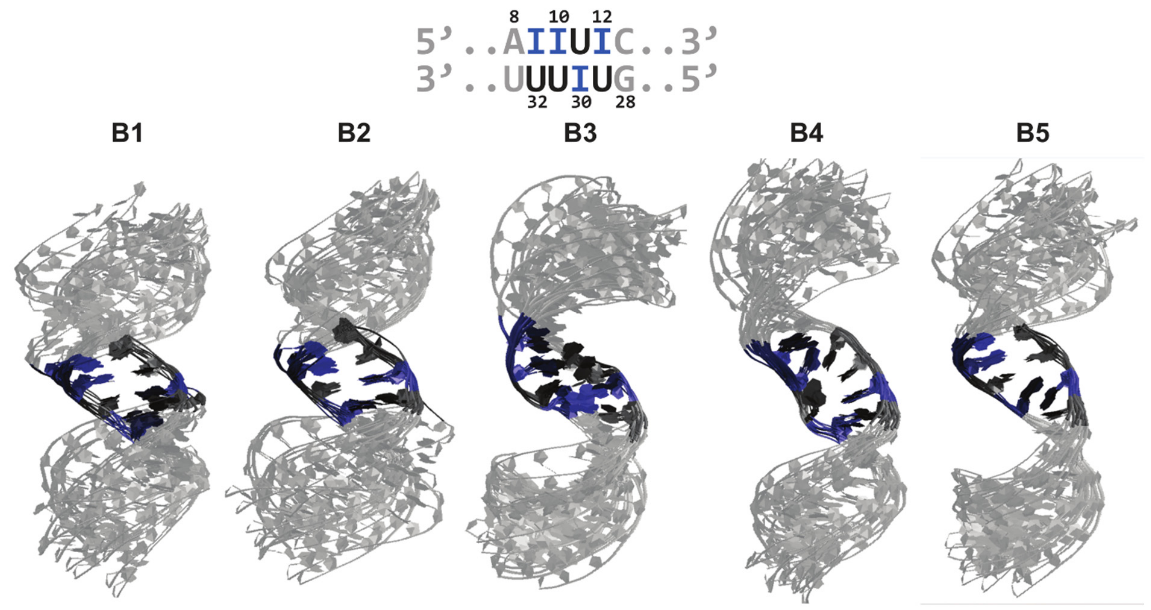

Figure S16: Overlays of individual structures from the five clusters (B1-B5) in respect to the central motif. The central motif is color-coded accordingly, with inosines in blue and uracils in black. The flanking helical regions are shown in light gray.

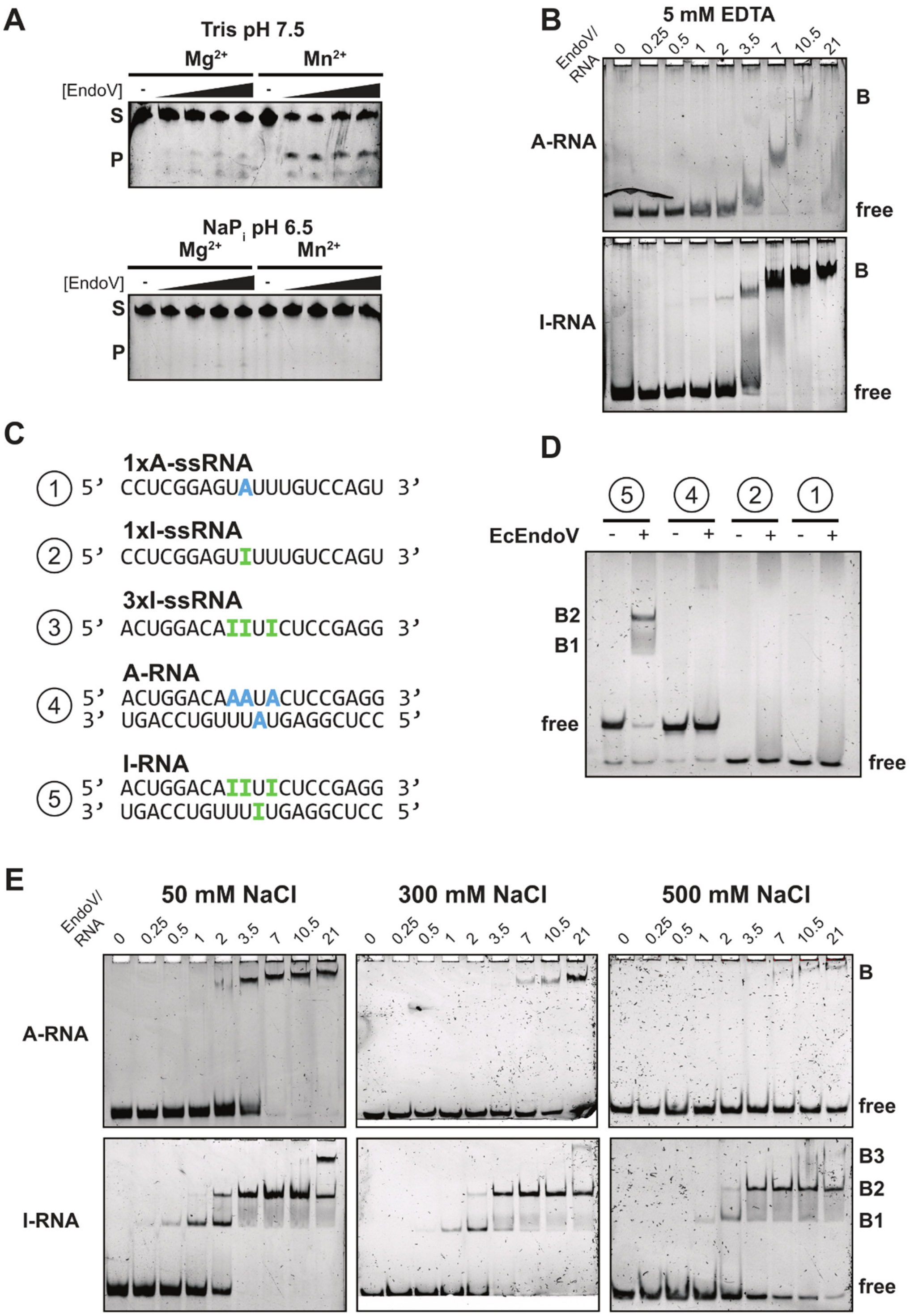

Figure S17: (A): Enzymatic cleavage assays under different buffer conditions. Increasing concentrations of EcEndoV have been added to a constant concentration of I-RNA under different buffer conditions. The enzyme is inactive for low pH conditions. (B): Effect of EDTA on the binding behavior of EcEndoV. To each reaction, 5 mM EDTA were added instead of bivalent cations. The binding species were separated on a 12% native PAGE. The gels were stained using 0.004% SYBR gold solution in TBE. Addition of EDTA disrupts the binding behavior of EcEndoV, showing the importance of bivalent cations as co-factors. (C): Different single-stranded and double-stranded RNA substrates with and without inosines. (D): EMSA

using the different substrates. EcEndoV was added at 3.5x molar excess to 200 nM RNA. After incubation on ice, the binding species were separated on a 12% native PAGE. The gel was stained using SYBR gold. Only I-RNA shows the complex bands B1 and B2 while the single-stranded substrate do not show detectable binding. This shows that binding to I-RNA does not occur to the individual separated strands. (E): Influence of the salt concentration on RNA binding. EMSAs were performed at 50, 300 and 500 mM NaCl using A-RNA and I-RNA. The binding species were separated on a 12% native PAGE and visualized by staining the gel with SYBR gold. Binding of A-RNA which occurs in an unspecific electrostatic manner, can be inhibited at high salt concentration. The same is observed for band B3 in I-RNA, whereas complex bands B1 and B2 remain visible. This shows that binding mode B3 is comparable to the binding of A-RNA. Furthermore, bands B1 and B2 arise from a highly specific interaction with the conformationally distinct I-RNA.
